# Cascading effects of drought in Xilin Gol temperate grassland, China

**DOI:** 10.1038/s41598-023-38002-2

**Published:** 2023-07-05

**Authors:** Jingzhao Ma, Jingmin Gao

**Affiliations:** 1 Collaborative Innovation Center on Forecast and Evaluation of Meteorological Disasters (CIC-FEMD)/Jiangsu Key Laboratory of Agricultural Meteorology, Nanjing University of Information Science and Technology, Nanjing, People’s Republic of China; 2 Longchuan Meteorological Bureau, Longchuan, 517300, Guangdong People’s Republic of China; 3 Emergency Management College, Nanjing University of Information Science and Technology, Nanjing, People’s Republic of China

**Keywords:** Environmental sciences, Hydrology, Natural hazards

## Abstract

In the context of global climate change, the cascading risk of compound natural hazards is becoming increasingly prominent. Taking Xilin Gol grassland as study area, we used the Mann–Kendall trend method, the maximum Pearson correlation coefficient method, and Partial least squares structural equations modeling to detect the characteristics of spatiotemporal pattern changes of the three types of droughts. The propagation characteristics and the cascade effects among the three types of droughts was also identified. The standardized precipitation evapotranspiration index, standardized evapotranspiration drought index, and soil moisture index were selected as indicators of meteorological drought, ecohydrological drought, and soil drought, respectively. The results show that the warm and dry trend in Xilin Gol grassland was obvious in the past 30 years. The seasonal propagation of different drought was prominent, with stronger spread relationships in summer. Persistent meteorological drought was more likely to trigger the other two types of droughts. The intensity and range both increased during the propagation from meteorological drought to ecohydrological drought. The cascade effect was differed in different time scales. The multi-year persistent climatic drought has an overwhelming cascade effect on soil drought and ecohydrological drought. For seasonal or annual drought, vegetation cover change has an amplifying or mitigating impact on the cascade effect, where soil moisture, evapotranspiration (ET), and their relationship all play important roles. In eastern areas with better vegetation cover, the reduction of vegetation in the early stage aggravated the cascading effect of meteorological drought to ecohydrological drought through reducing ET. In the northwestern sparsely vegetated areas, ET was mainly influenced by meteorological factors, and the cascade effect of meteorological factors to ecohydrological drought was more obvious than that of soil drought.

## Introduction

In the context of global changes, the suddenness, concurrency, unpredictability and associated risks of various natural disasters are becoming increasingly prominent^1^. Previous studies have shown that greenhouse gas emissions will contribute to the simultaneous or sequential occurrence of multiple meteorological disasters, leading to an increase in extreme weather events and longer periods of drought^2^. This will have a more profound impact on ecosystems, with a strong and rapid response to grassland ecosystems in particular. Droughts are highly destructive natural disasters and, typically, can be classified into four types, including meteorological drought, agricultural drought, hydrological drought and socio-economic drought. In addition, another form of drought called “Ecohydrological drought”. When ecosystems are affected by drought, the availability of water for vegetation is reduced, which affects ecosystem services and triggers feedback from natural and/or human systems. Such droughts are called Ecohydrological droughts^3^. ET is a critical variable for enhancing and sustaining ecological and water productivity, it also plays a critical role in hydrologic balances, climate change studies, plant physiological functions, and hydrology and ecosystem relationships^4^. Meteorological drought can affect the ecohydrological process, this means that meteorological drought and ecohydrological drought are interrelated^5^.

Droughts initially occur as a result of chronic precipitation shortages, and it is possible for one type of drought to have an effect on other components of the hydrological cycle under prolonged occurrences and then convert to another type of drought^6^. This spatial and temporal relationship of multiple drought type transitions is known as drought propagation^7^, also known as cascading effects between droughts^8^. It is found that meteorological drought is the main triggering factor for other drought types^9^. A long-term meteorological would probably lead to a decrease in surface runoff or groundwater levels, which leads to hydrological drought. It may also lead to soil moisture deficit, which results in soil drought^10^. Socio-economic drought occurs when the supply of water cannot meet demand^11^. A shortage of soil water content will affect the processes of surface evapotranspiration or runoff generation. Therefore, soil drought may also have an impact on the occurrence of hydrological drought.

A large number of previous studies have tended to focus on the risks posed by a single type of drought, which may overlook the cumulative or cascading effects of meteorological droughts with other types of droughts, resulting in an incomplete assessment of the impacts of climate change^12,13^. In recent years, the propagation between different types of drought hazards is gradually getting attention. Some scholars have mainly focused on the use of different drought indices to identify changes in the characteristics of various drought events (frequency, duration, severity, and range of impact of droughts) and on the relationships between different drought types. For example, Gu et al.^14^ analyzed the relationship between meteorological drought and hydrological drought in the Jinsha River Basin, Yellow River Basin, and Jialing River Basin in China, and found that strong and robust correlations existed between meteorological and hydrological drought hazards. Ding et al.^15^ showed that in different climatic zones of China, the propagation relationship between meteorological and hydrological droughts was weaker in arid environments than in wet environments, and the propagation relationship between the two types of droughts was stronger in summer and autumn than in spring and winter. Chen et al.^16^ found that the propagation time from meteorological to agricultural droughts, and that from agricultural to hydrological droughts, showed remarkable seasonal characteristics in the Luanhe River basin in China. Yang et al.^17^ found that the sequence of drought propagation varies by altitude, and altitude plays a key role in the spatial and temporal differentiation of drought propagation in plateau mountains. Ding et al.^18^ found that different climatic regions had different major drivers of drought, and soil moisture (SM) was usually the most important driver of agricultural drought in all climate regions. It can be noted that the propagation relationship of drought varies according to the differences in land cover, topography and climatic zones, and its propagation process is intricate and complex. In general, although some studies have found connections between different types of droughts, most of them have discussed the propagation of drought using simple correlation methods. Due to the complexity of the causes and mechanisms of different types of droughts, simple correlation analysis cannot clearly indicate whether there is a real causal relationship between them and how they are influenced by the changing environment, and thus cannot well detect and explain the "cascade effect" of drought.

Grasslands consist of many interconnected biophysical systems. It provides a range of other ecosystem service functions in addition to livestock products for humans^19^. If one of these systems breaks down, it can have a huge impact on the entire grassland system, which in turn can lead to devastating and irreversible changes^13^. The process of formation, outbreak and development of grassland hazards is often very complex. Spatially, it is characterized by the interconnection and combination of multiple hazards in the same area. Global warming and human activities have been leading to an increase in grassland droughts. Frequent meteorological droughts accelerate the degradation of grassland ecosystems by altering the water and energy balance of grassland surfaces and affecting ecohydrological processes^20^. Previous studies have shown that compared to single disasters, grassland meteorological drought disasters significantly amplify the disaster situation by evoking soil and hydrological hazards^21^. This is not only extremely detrimental to grassland animal husbandry and restricts local economic and social development, but also has an important impact on the carbon source and sink function of grassland ecosystems and exacerbates grassland degradation^22,23^.

The Xilin Gol grassland in Inner Mongolia belongs to the temperate grassland, which is the largest livestock production base in China. However, drought is one of the main natural disasters that occur frequently due to the uneven spatial and temporal distribution of low precipitation in the region. The frequent occurrence of persistent and seasonal droughts in this region in recent years, combined with the decrease in heavy rainfall events, has led to increased soil drought, reduced grassland biomass, and increased grassland degradation^20,22,24^. However, it is uncertain whether there are causal relationships and feedbacks between these hazards and how the disaster amplification effects of drought are transmitted. Using the Xilin Gol grassland as study region, specifically, we were to explore the following questions: (1) Detection of spatial and temporal trends of meteorological drought, ecohydrological drought and soil drought and analysis of their spatial and temporal variability; (2) Effects of biophysical drivers on three types of droughts in different periods; (3) Identify the seasonal transmission characteristics of three kinds of droughts and their cascading effects.

## Results

### Annual trends of drought indices

**Figure 1:**
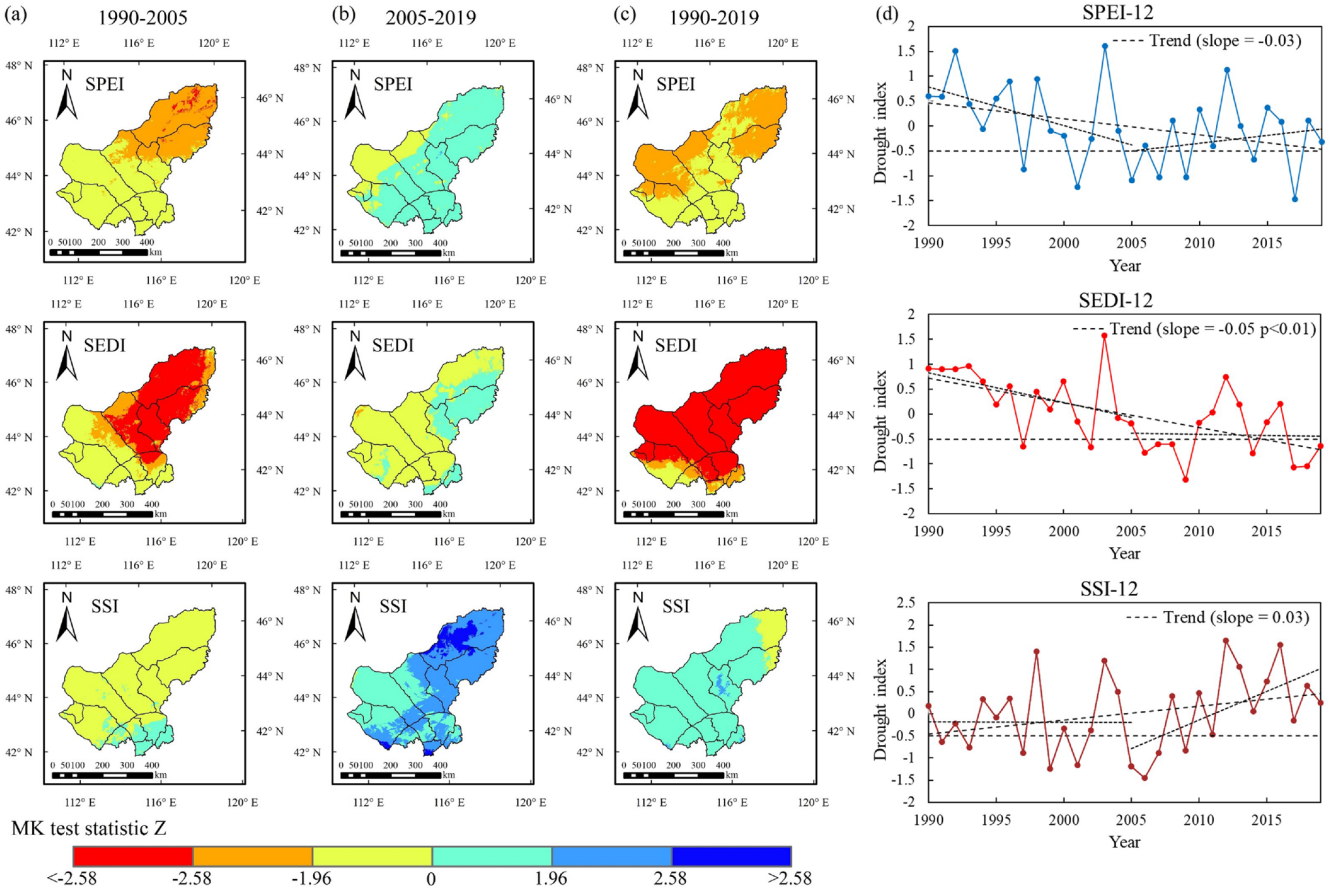
Spatial annual trend variation of three drought indices at different time periods (**a**) 1990–2005; (**b**) 2005–2019; (**c**)1990–2019; (**d**) Temporal annual trend variation of three drought indices. (The figure was generated by ArcGIS 10.6 software, https://desktop.arcgis.com/en/).

**Figure 2:**
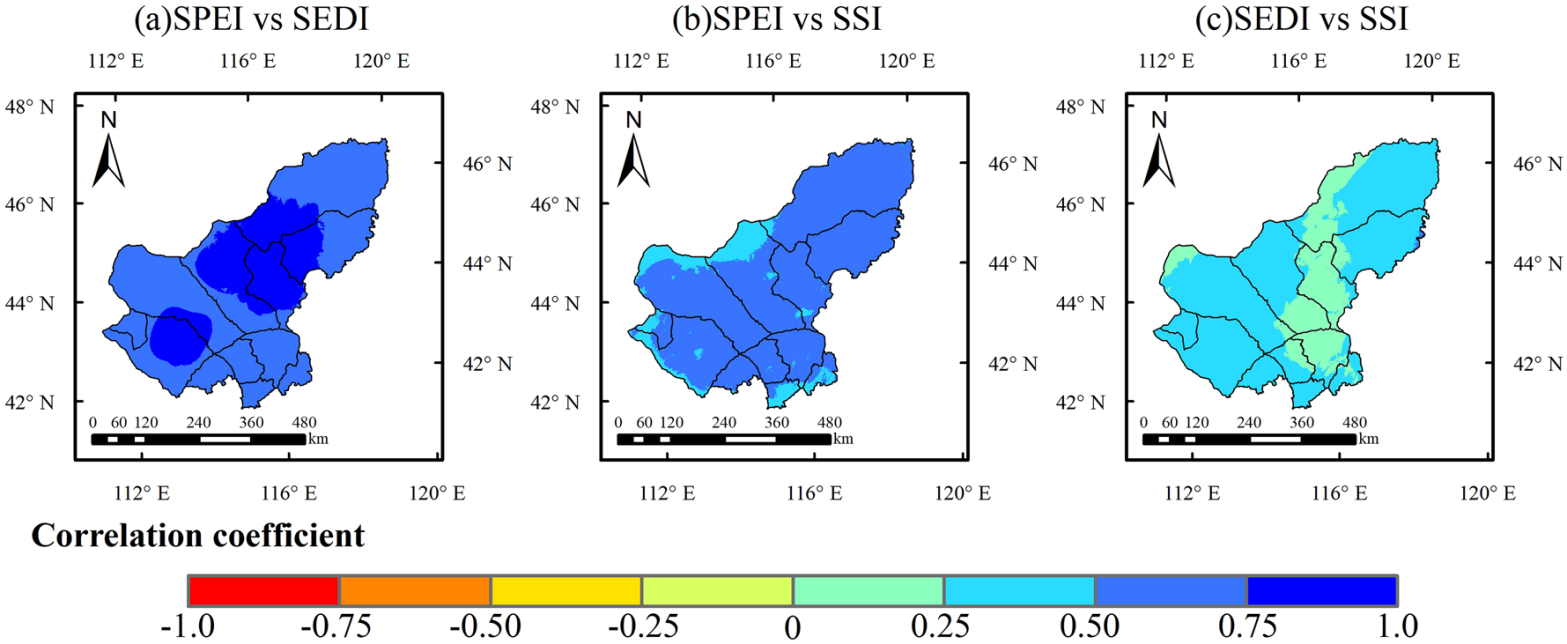
Correlation of Annual meteorological drought, ecohydrological drought and soil drought (1990–2019). (The figure was generated by ArcGIS 10.6 software, https://desktop.arcgis.com/en/).

Based on the trend test and correlation methods, Fig. 1a–d shows the trends of SPEI-12, SEDI-12 and SSI-12 in temporal and spatial scales, respectively. Figure 2 shows the correlation coefficients between the three drought indices. The result show that in the last 30 years, the Xilin Gol grasslands have experienced a long-term warm drying process, with the most pronounced drought (DI $\le$ -0.5) at the beginning of the twenty-first century (2000–2009) (Fig. 1d). The three drought indices began to gradually increase after 2005, and the drought situation was alleviated. In particular, soil moisture (SSI-12) alleviated more significantly after 2005 (Fig. 1b,d). On the 30-year scale, the trend of both SPEI-12 and SEDI-12 changes showed a decreasing trend (Fig. 1c,d). It is noteworthy that there is an enhanced degree and expansion of the propagation of meteorological drought (SPEI) to ecohydrological drought (SEDI) (Fig. 1c). In different time periods, the trend of all three drought indices in most regions of the grassland was decreasing in the early period of increased drought (1990–2005), with the most significant decrease in ecohydrological drought in the central and eastern regions (Fig. 1a). During the late period of drought persistence (2005–2019), SPEI-12 showed a trend of non-significant increase (wetting) in most regions, and the wetting trend of soils was evident in the southeast (Fig. 1b).

The correlation analysis of different drought types showed that meteorological drought was significantly correlated with ecohydrological drought and soil drought (Fig. 2a,b), but ecohydrological drought (SEDI) was not significantly correlated with soil drought (SSI) (Fig. 2c). Similar characteristics were found in the early period of increased drought (1990–2005) (Fig. S1a), where the main factor affecting ecohydrological drought was meteorological drought. The correlation between SSI and SEDI was significantly stronger in the later stages of drought persistence (2005–2019), especially in regions where the correlation between meteorological drought and ecohydrological drought was high (Fig. S1b, r > 0.5). Meteorological drought and soil drought jointly influence ecohydrological drought in most regions (Fig. S1b).

### Effects of biophysical drivers on droughts

**Figure 3:**
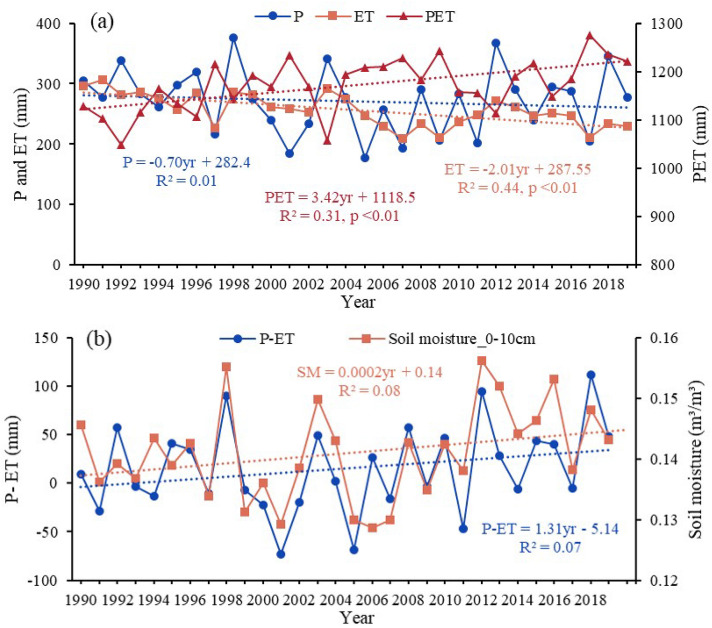
Spatial trend distribution of MK with different drought indices and correlations between different drought indices.

**Figure 4:**
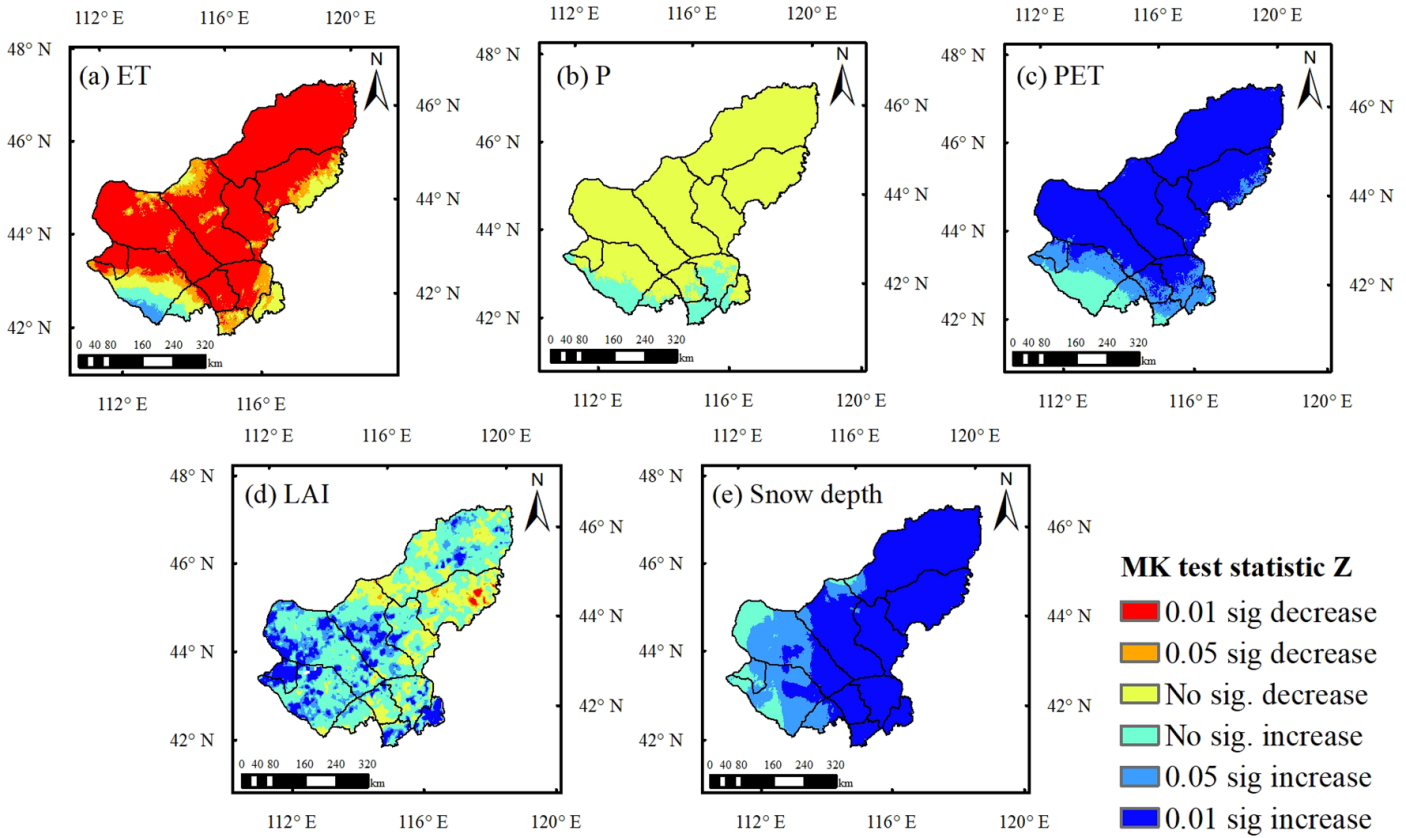
Spatial trend distribution of biophysical drivers (**a**)–(**e**). (1990–2019). (The figure was generated by ArcGIS 10.6 software, https://desktop.arcgis.com/en/).

**Figure 5:**
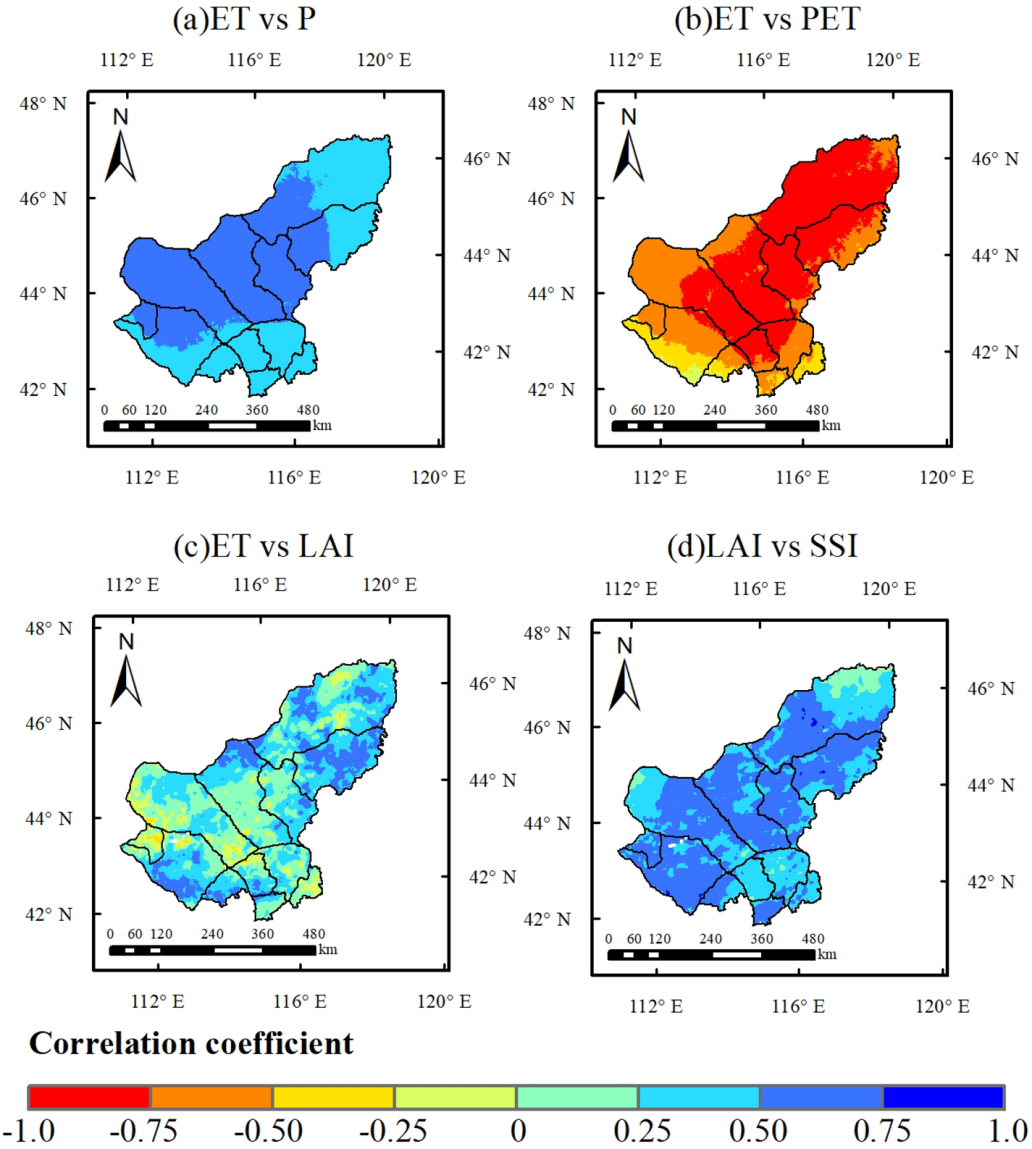
Correlation between different biophysical drivers. (**a**) ET vs. P, (**b**) ET vs. PET, (**c**) ET vs. LAI, and (**d**) LAI vs. SSI during 1990–2019. (The figure was generated by ArcGIS 10.6 software, https://desktop.arcgis.com/en/).

Evapotranspiration (ET) can be considered as a central component of the terrestrial water cycle, which has a significant impact on the availability and use of water resources. In addition, vegetation and precipitation are the dominant factors for ET trends. Snow depth in winter is also one of the reasons affecting ET and soil moisture in the study area. Therefore, we selected five potential influencing factors driving drought (Fig. 4). Figure 3a shows the annual temporal trends of precipitation (P), evapotranspiration (ET) and potential evapotranspiration (PET). It shows that there is a continuous significant increase in potential evapotranspiration PET in the study area over the last 30 years, as well as a non-significant decrease in annual precipitation P. This result show that meteorological drought continued to intensify, which resulted in restricted soil moisture availability. As a result, ET showed a significant reduction trend, i.e., an aggravation of ecohydrological drought (Fig. 3a). Combining the spatial trends (Fig. 4) with the correlation plots (Fig. 5), it is shown that ET is highly correlated with P and PET in most regions (Fig. 5a,b, r > 0.5). ET was more significantly affected by PET, and the two were significantly negatively correlated in most of the regions. Together with the non-significant decrease in P (Fig. 4b), this resulted in a significant decreasing trend in ET in most of the regions (Fig. 4a).

In addition, we also found that the significant decrease in ET was also associated with vegetation deterioration (Fig. 5c). The significant decrease in ET in the central-eastern region occurred mainly in the early stage (Figs. 3a,S4a). Further, the decrease in P, increase in PET, and deterioration in vegetation LAI were all influential factors in the significant decrease in ET (Figs. S4,S5), i.e., the degradation of vegetation in the east-central region in the early period aggravated the effect of meteorological drought in reducing ET (increased ecohydrological drought) (Figs. 4d,5c). In the later stages, P rebounded (Fig. 3a), while vegetation LAI improved in most areas, especially in the east (Fig. S6, S7). Therefore, the decreasing trend of ET becomes slower (Fig. 3a), but still maintains low values. For the western region, ET was not significantly correlated with LAI in the last 30 years (Fig. 5c), i.e., although the western vegetation LAI improved significantly (Fig. 4d), ET decreased significantly (Fig. 4a). This indicates that ET in the western sparsely vegetated areas is mainly influenced by meteorological factors.

**Figure 6:**
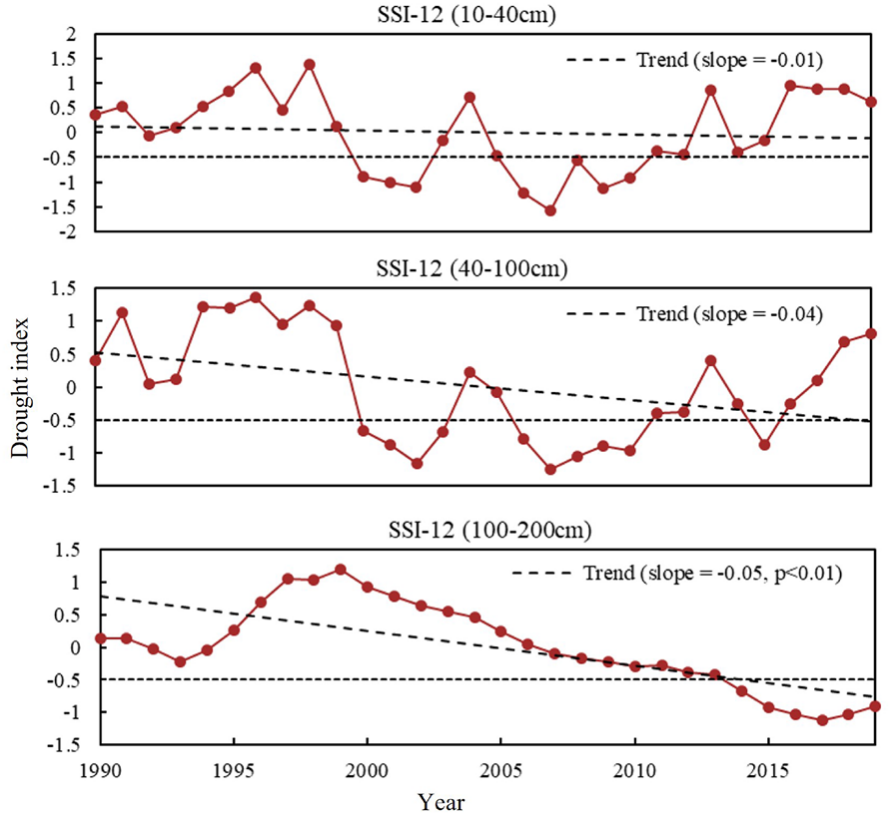
Annual trends of soil drought (SSI) in (**a**) 10-40 cm, (**b**) 40-100 cm, and (**c**) 100-200 cm.

We found an opposite trend of P and surface soil moisture (SM) (Fig. 3a,b). However, seasonally, P and surface SM trends were consistent. Both of them show a decreasing trend in summer and an increasing trend in spring, autumn and winter (Figs. S2a,S2b). Therefore, we speculate that although P is the dominant factor in the interannual fluctuations of surface SM, it may not be the cause of the increase in surface SM. Further analysis revealed that the severe soil drought in the previous period (1990–2005) tended to be the years when ET was greater than P. Soil drought was alleviated in the later period (2005–2019), mainly due to the improvement in P. ET stabilized but remained low, which led to an increase in P-ET and an increase in soil surface water storage (Fig. 3b). The water balances between precipitation input and ET output reflect soil water recharge. The contrasts between P and ET determine the soil moisture dynamics and thus the water available for plant use. In addition, we also found a significant positive correlation between most surface SM and LAI (Fig. 5d), indicating that grassland vegetation is sensitive to surface SM. However, it is noteworthy that the deep SM has decreased very severely in the last 30 years (Fig. 6), indicating that the long-term persistent drought in the study area has led to the lack of water supplement and continuous deficit in the soil, which in turn has caused a decrease in the deep SM content. In the long term, the cascading effect of persistent drought poses a greater threat to grassland ecosystems.

We further found a significant upward trend (p < 0.05) in snow depth in the study area (Fig. 4e). Seasonally, both spring temperature and winter snow depth showed a significant upward trend (Fig. S3a). In the recent 30 years, the winter snow depth showed an increasing trend (UF > 0) and the mutation point was in 1998 and started to increase significantly (p < 0.05) in 2001 (Fig. S3b). In contrast, spring surface SM showed a non-significant decreasing trend (p > 0.05) until 1998, and an increasing trend started in 1998 and the mutation point was in 2001 (Fig. S3c). Therefore, in the later period, we speculate that the abrupt rise in snow depth in winter and the increase in temperature in spring may be one of the reasons for the increase in snowmelt and soil moisture in spring.

### Seasonal propagation between three drought indices

**Figure 7:**
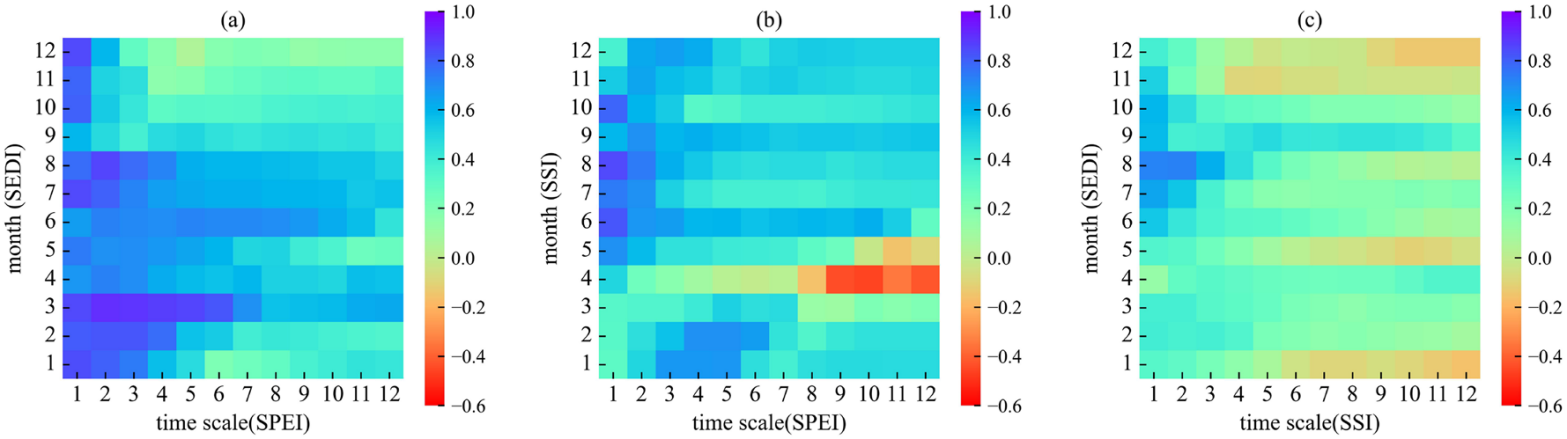
Seasonal variability in drought propagation time. (**a**) SPEI-n and SEDI-1; (**b**) SPEI-n and SSI-1; (**c**) SSI-n and SEDI-1. (The figure was generated by python 3.9, https://www.python.org/).

The correlation coefficient of the monthly scale (1–12 month) drought index calculated using the MPCC method shows that the propagation time from meteorological drought to ecohydrological drought is around 1 month (r > 0.6) (Fig. 7a). The propagation relationship from meteorological drought to soil drought was significantly seasonal and cyclical (Fig. 7b). Its high correlation (r > 0.6) was mainly concentrated in the growing season (April-September), with a propagation time of less than 1 month. The average propagation time in winter is about 4 months, mainly because the response of soil drought to meteorological drought becomes faster in this region during the growing season when precipitation and temperature increase. Similarly, the correlation between soil drought and ecohydrological drought was highest (r > 0.5) in summer (June–August) (Fig. 7c). Their propagation characteristics are also distinctly seasonal, with an average propagation time of less than 1 month and lower correlation coefficients during the vegetation dormancy period in winter. Similarly, the correlation between soil drought and ecohydrological drought was highest (r > 0.5) in summer (June–August) (Fig. 7c). Its propagation characteristics are also distinctly seasonal, with an average propagation time of less than 1 month, and a lower correlation coefficient in the winter when vegetation is dormant. It can be concluded that the propagation time of different droughts is shorter in the warm and humid season with more precipitation and higher temperature, and longer in the cold season. Therefore, the occurrence of meteorological drought may lead to soil drought caused by the lack of soil moisture in the short term, and soil drought further triggers ecohydrological drought. The three drought types form a cascade effect.

### Cascading effects between three droughts

The model path analysis showed that meteorological drought in different regions of the Xilin Gol grassland had significant effects (*p* < 0.05) and strong causal relationships on both soil drought (f^2^_east_ = 0.497, f^2^_south_ = 0.202, f^2^_northwest_ = 0.335, f^2^_center_ = 0.270) and ecohydrological drought (f^2^_east_ = 1.170, f^2^_south_ = 0.639, f^2^_northwest_ = 0.765, f^2^_centre_ = 1.209) in the recent 30 years (Table S10, S14), indicating that meteorological drought is more likely to cause the occurrence of the other two types of drought.

**Figure 8:**
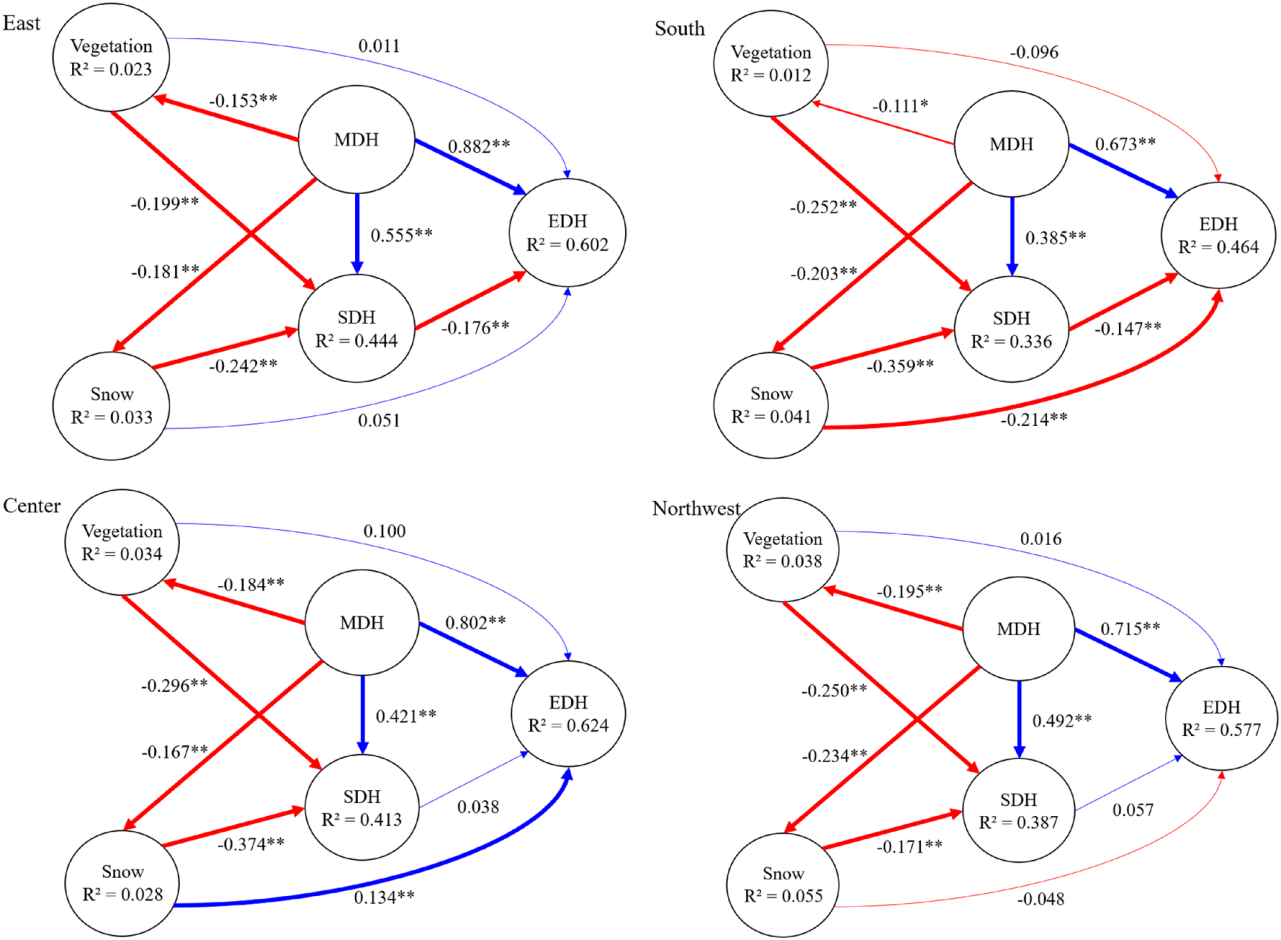
A causal path model between different drought types in East, South, Northwest, Center. Note: MDH, SDH and EDH represent meteorological drought hazard, soil drought hazard and ecohydrological drought hazard, respectively. Blue represents positive path coefficients; red represents negative path coefficients. The thickness of the line represents significance, *p < 0.05, **p < 0.01.

The cascade effect of different regions further confirmed our previous speculations. The combined effect of exogenous latent variables (MDH, SDH, vegetation, snow) on EDH (ecohydrological drought) was stronger (Table S9, R^2^ = 60.2%) in the eastern regions with better vegetation cover (forest and meadow steppe). In particular, there was a significant interaction between soil drought and ecohydrological drought, but the causal relationship was weak (Table S10, f^2^_east_ = 0.043). Furthermore, the negative value of the path coefficient (Fig. 8, PC_east_ = −0.176) indicates that soil drought in this region does not necessarily initiate ecohydrological drought. On the contrary, the reduction of annual ET may increase annual soil water storage and alleviate soil drought, which confirms our previous speculation (Fig. 5b). In addition, we found that eastern vegetation has a mediating effect on ecohydrological drought (Table S10, VAF = 76.1%), i.e., vegetation affects the cascade effect from meteorological drought to ecohydrological drought. In other words, worse (or improved) vegetation aggravates (or mitigates) the effect of meteorological drought in reducing ET (aggravating ecohydrological drought).

The combined explanation of EDH by exogenous latent variables (MDH, SDH, vegetation, snow) was also stronger in the northwestern sparsely vegetated regions (Table S13, R^2^ = 57.7%). In this region, the causal relationship between meteorological drought and ecohydrological drought was significant. Different from the eastern region, the path coefficient from soil drought to ecohydrological drought was low (Fig. 8, PC_northwest_ = 0.057) and the causal relationship was not significant. However, The causal relationship from vegetation to ecohydrological drought was also not significant (Fig. 8, PC_northwest_ = 0.016). Soil moisture was less in the northwest and vegetation was sparser. ET was mainly influenced by meteorological factors, i.e., the main influence of ecohydrological drought in the region was meteorological drought (Table S14, f^2^ = 0.762). The effects of both vegetation and soil drought on ecohydrological drought were not significant.

The effects of meteorological drought on vegetation changes were weak in the whole study region (0.012 ≤ R^2^ ≤ 0.038, Table S10, S14). It may be due to the fact that anthropogenic factors were the main reason affecting vegetation improvement in recent years (e.g., the policy of returning grazing to grassland). As shown by the path coefficient PC (Fig. 8), vegetation and snow also had a significant negative effect on soil drought hazard, i.e., both vegetation and snow depth played a role in alleviating soil drought.

In conclusion, meteorological drought can either directly initiate ecohydrological drought, or initiate ecohydrological drought by causing soil drought, or affect soil moisture by causing ecohydrological drought. This depends mainly on whether the dominant factor affecting ET is meteorological (e.g., PET or P) or subsurface (e.g., vegetation). The former mainly causes soil drought by affecting soil evaporation, which in turn causes ecohydrological drought; the latter mainly affects ecohydrological drought by contributing plant transpiration, which in turn affects soil moisture.

## Discussion

### Propagation characteristics of different drought types

In arid and semi-arid regions, the actual evapotranspiration (ET) is mainly determined by precipitation (P)^25^ and usually the ET is almost equal to the P^26^. It is difficult to generate runoff in water-limited regions (PET > P), Since the PET is much larger than the P, most of the P returns to the atmosphere as ET and only a small amount is stored in the soil. In this study we found that in arid and semi-arid temperate grassland regions, the response time of ecohydrological drought to meteorological drought was rapid and without obvious seasonality (Fig. 7a). However, the propagation characteristics of the two propagation chains (Fig. 7b,c), meteorological drought to soil drought and soil drought to ecohydrological drought, had a distinct seasonality. The propagation of meteorological drought and soil drought was shorter in spring and summer. Ecohydrological drought could be mitigated by spring snowmelt, in which it was not sensitive to soil drought. Thus, there was a stronger relationship between soil drought and ecohydrological drought in summer than in spring. This was similar to the study by Ding et al.^18^, who found that in northern China (arid and semi-arid regions), the propagation from meteorological drought to agricultural drought had a significant seasonality, with a stronger relationship from agricultural to hydrological drought in summer than in spring. It is perhaps not surprising that the water demand of vegetation increases with the coming of spring. Precipitation in the study region is concentrated in the growing season (April–September), where summer is the peak season for vegetation. If meteorological drought was caused by insufficient precipitation during this period, it would lead to a lack of soil water, and coupled with the fact that evaporation of soil water would be accelerated in the high temperature environment in summer, this situation would more easily lead to soil drying. In these conditions, vegetation also has to consume soil water storage to maintain transpiration due to lack of precipitation^22^. Overall, we concluded that there was a stronger propagation relationship between different drought types in temperate grasslands in summer.

### Influence of vegetation on drought cascade effects

We found that meteorological drought had a strong causal effect on soil drought and ecohydrological drought in temperate grasslands. This means that meteorological drought can easily generate cascading effects. Different grassland types respond differently to drought^27^, which in turn affects the cascading effects of drought. Significant climatic differences between the eastern and western areas of the study region resulted in distinct differences in vegetation types. The eastern part is meadow steppe and forest, and the southern part is typical grassland and cropland, with high LAI in both areas. Therefore, transpiration of vegetation in the east and south is the main way of water exchange between the surface and the atmosphere, i.e., transpiration of vegetation dominates ET. Soil moisture is connected to atmospheric moisture through direct evaporation and vegetation transpiration^28^. Jung et al.^29^ found that ET increases during drought due to increased atmospheric water demand and that increased ET decreases soil moisture. We found that the cascade effects of drought in the eastern and southern parts of the study region were similar to this finding. On the one hand, when meteorological drought occurs, the well-developed root system of vegetation in a water-scarce environment absorbs water from the soil to maintain its transpiration (SM decreases), which makes the risk of ecohydrological drought decrease (ET increases). On the other hand, meteorological drought reduces soil water supply, ET usually decreases, evaporation from bare soil decreases, plant stomata close and transpiration is weakened, which directly leads to soil drought and ecohydrological drought.

Different from the eastern and southern regions, the central part is a typical grassland and the northwestern part is a desert grassland with lower LAI in both areas. In general, better vegetation cover can increase soil infiltration^30^ and improve soil water storage capacity. The less vegetation cover in the central and northwestern parts leads to a weaker soil water storage capacity. Precipitation in arid and semi-arid regions is the main influence on soil moisture and evapotranspiration, which is quickly returned to the atmosphere as evaporation after reaching the surface. Thus, in the central and northwestern part of the study region, meteorological drought was the main cause of soil drought and ecological drought, while the causal relationship between soil drought and ecohydrological drought was weak. In addition, due to the low LAI of vegetation and more exposed surface soil in this region, the solar radiation energy reaching the surface makes the surface evaporation more intense, and the contribution of surface evaporation to evapotranspiration is higher than that of transpiration by vegetation. In contrast, the enhanced evapotranspiration caused by the increase of LAI in the central region in recent years may cause the reduction of water available at the surface. Furthermore, recent studies have found that the sensitivity of LAI to soil moisture increases significantly in many arid and semi-arid regions around the globe^31^. Similarly, our results suggest that the trend of LAI Fig. 4d, S4d, S6d) in most regions was consistent with soil moisture (Fig. 1a–c).

### Influence of other factors on drought cascade effects

The cascading effect of drought is a complex process, which involves ecological environment, social economy and culture and so on. The propagation of drought is not only related to climatic factors, but also influenced by human activities. For example, the implementation of ecological and environmental construction policy measures such as "returning grazing to grassland", "natural grassland protection" and "fencing" in the study region has improved the vegetation cover^20^, which also had an impact on the propagation of drought. In addition, human disturbances due to overgrazing, mining, and overuse of groundwater also have an impact on drought changes. Wang et al.^32^ found that continued increases in domestic and industrial water use caused severe losses of surface water, which in turn prolonged the response time of hydrological drought to meteorological drought. Human activities significantly alter surface hydrological processes, mainly including evapotranspiration, water infiltration, and soil water storage, which in turn affect the development of drought^33^. Therefore, in future studies, we should further focus on the influence of human factors on the drought cascade effect.

## Conclusions

In this study, the Mann–Kendall trend method and the maximum Pearson correlation coefficient (MPCC) method were used to detect the characteristics of spatial and temporal pattern changes of meteorological drought, ecohydrological drought and soil drought in the study area during 1990–2019 and their propagation characteristics in different seasons, using the temperate Xilin Gol grassland in Inner Mongolia as the study area. The cascading effects among the three droughts were quantified and analyzed using PLS-SEM considering the effects of vegetation and snow depth. The results showed that the warm and dry trend of Xilin Gol grassland was obvious during the last 30 years, especially during 1990–2005. The drought situation was alleviated but persisted after 2005. The intensity and range of meteorological drought gradually increased during its propagation to ecohydrological drought. The propagation of different drought types had seasonal characteristics, and the relationship between the propagation of different drought types was strong in summer. PLS-SEM cascade analysis further showed that warm drying had a significant cascade effect (p < 0.05) on both soil drought and ecohydrological drought, i.e., persistent meteorological drought was more likely to initiate the other two types of droughts. This cascade effect was enhanced or reduced by winter snowpack and vegetation conditions.

We suggest that there are differences in drought cascade effects on different time scales, such as seasonal drought, annual drought and multi-year persistent drought cascade effects. Seasonally, on the one hand, soil moisture supply is restricted during meteorological drought, ET decreases, evaporation from bare soil is reduced, plant stomata are closed, and transpiration is weakened. On the other hand, under the combined effect of abnormally high temperature and precipitation deficit, ET may also increase with the increase of atmospheric water demand, resulting in continuous local soil moisture deficit, thus accelerating the depletion of water resources and causing destructive sudden drought events, which intensify ecosystem stress. On an annual scale, the reduction in vegetation LAI may lead to a reduction in plant transpiration. In the case of no decrease or even an increase in precipitation, ET reduction may increase soil water storage and alleviate soil drought. Persistent multi-year climatic drought not only leads to stunted vegetation growth and reduced ET, but also results in a continuous soil water deficit, which in turn leads to a decrease in deep soil water content. In the long term, the cascading effect of persistent drought poses a greater threat to grassland ecosystems.

In addition, the cascade effect of drought varies across regions. In this study, in the eastern and southern regions with better vegetation cover, the deterioration of vegetation aggravated the effect of meteorological drought in reducing ET (aggravating ecohydrological drought), i.e., vegetation affects the cascade effect from meteorological drought to ecohydrological drought. However, in the central and northwestern areas with sparse vegetation, ET was mainly influenced by meteorological factors, the role of vegetation was not significant, and the cascade effect between soil drought and ecohydrological drought was weak. In conclusion, the influence of multi-year persistent meteorological drought on the cascade effect is decisive. However, for the cascade effect of seasonal or annual drought, vegetation has an amplifying or mitigating effect on the cascade effect of meteorological drought, in which soil moisture, ET, and the relationship between them both play a highly important role.

## Methods

### Study area

**Figure 9:**
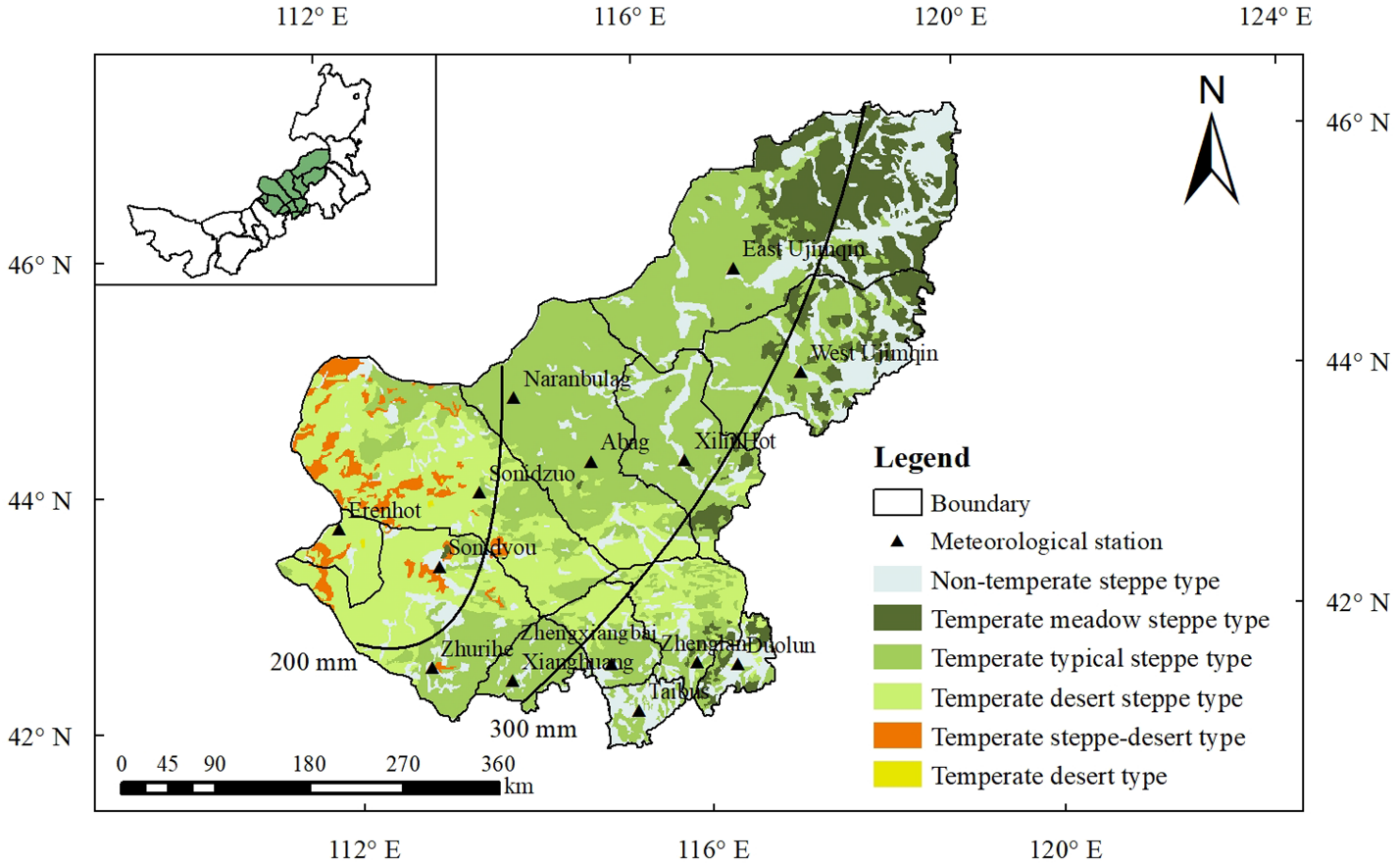
The study area and meteorological observation sites in Xilin Gol region. The lines are annual precipitation during 1990–2019. (The figure was generated by ArcGIS 10.6 software, https://desktop.arcgis.com/en/).

The Xilin Gol Grassland is located in the central part of the Inner Mongolia Autonomous Region of China. The grassland area is about 19.2 × 10^4^ km^2^, accounting for more than 95% of the total area. It is a native temperate grassland and is the largest livestock production base in China. It belongs to a typical temperate continental climate mainly characterized by aridity, cold, high wind, low precipitation and high evaporation. The average annual temperature is between 0 and 3 ℃, and the annual snowfall is about 17 mm, which mainly occurs in autumn and winter (from November to March). Annual precipitation is between 200 and 400 mm, mainly occurs in summer (June–August). Precipitation gradually decreases from southeast to northwest, and overall precipitation is low. Evaporation is higher than precipitation in most areas, and the uneven spatial and temporal interannual distribution of precipitation leads to frequent regional droughts. The Xilin Gol grassland is dominated by high plains and is an area with a variety of landforms, with high terrain in the south and low terrain in the north. The eastern and southern parts are mostly low hills, while the western and northern parts have flat topography, and the elevation of about 752–1876 m. The vegetation in the region is diverse, mostly highland grassland, with the main grassland types from east to west being meadow grassland, typical grassland, desert grassland, and sandy land in the south (Fig. 9). The growing season of the vegetation in this area is from April to September, and the peak growing season from June to August.

### Data

The daily meteorological data at 14 Meteorological stations across the Xilin Gol during the period of 1990–2019 obtained from the China Weather Data Website (http://data.cma.cn). The dataset includes precipitation (P, mm), relative humidity (%), wind speed (m/s), sunshine hours (h), daily average temperature (°C), daily maximum and minimum temperature (°C), and daily average water vapor pressure (kPa). The meteorological data are processed into monthly scales and interpolated to raster data with a spatial resolution of 1 km using the ANUSPLIN method for subsequent calculations. Anusplin is a tool for interpolating multivariate data using ordinary thin disk and partial thin disk spline functions, which has the advantages of high resolution and small interpolation error for time series meteorological data^34^. Daily ET during 2011–2012 in Xilinhot station was estimated by the weighing lysimeter method for validation. The effective ET area of the lysimeter was 4.0 m^2^ with a 2.6 tall, undisturbed soil column. The condensation of gaseous water was ignored when calculating ET, and the data during rainy periods were removed. The measurement accuracy of the lysimeter was 0.1 mm. The data were collected automatically and recorded every hour. Daily ET data were checked for consistency for rainy dates when problems occurred most often. The daily ET data was used to verify the subsequent estimated evapotranspiration.

In this study, we used monthly-scale soil moisture (0–10 cm, 10–40 cm, 40–100 cm, 100–200 cm) and monthly-scale snow depth data simulated by the Noah model of GLDAS-2 provided by the Global Land Data Assimilation System (GLDAS, in kg/m^2^ and m, with a spatial resolution of 0.25) for soil drought index calculation and snow accumulation analysis. To unify the study years, we selected monthly data values of soil moisture and snow depth from GLDAS 2.0 (1990–1999) and GLDAS 2.1 (2000–2019) and converted soil moisture and snow depth units to m^3^/m^3^ and mm, respectively. The data were interpolated to a 1 km spatial resolution grid using the ANUSPLIN method for subsequent raster data calculations. Leaf area index LAI data from 1990–2019 were obtained from the GLOBMAPLAI V3 product with a temporal resolution of 8 days and a spatial resolution of 0.08°^35^ (Table 1).

**Table 1:** Dataset used in this study.

Dataset	Parameters	Resolution	Period	Source
Meteorological station data	Precipitation (mm), relative humidity (%), wind speed (m/s), sunshine hours (h), maximum and minimum temperature (°C), and water vapor pressure (kPa)	Daily	1990–2019	http://data.cma.cn
Measured evapotranspiration	Evapotranspiration (mm)	Daily	2011–2012	Xilinhot station
GLDAS Ver. 2.0 and 2.1	0–10 cm, 10–40 cm, 40–100 cm, 100–200 cm Soil moisture, Snow depth	Monthly 0.25° × 0.25°	1990–2019	https://disc.sci.gsfc.nasa.gov
GLOBMAPLAI V3	Leaf area index, LAI	8 days 0.08° × 0.08°	1990–2019	Liu et al. 2012

### ET estimation using modified Granger and Gray Method

In the Bouchet (1963) hypothesis, there is a complementary relationship between Evapotranspiration (ET) and Potential evapotranspiration (PET), with Wet-environment evapotranspiration (ETw) is equal to PET and ET under saturated conditions (Eq. (1)). ETw means that if the soil plant surface is wet enough, ET can approach its potential value PET. Equation (1) indicates that PET decreases with increasing ET. In other words, as the surface dries, the ET decreases, resulting in lower humidity and higher temperature of the surrounding air, and therefore PET will increase. Among the various complementary relationship (CR) methods to estimate ETw and ET, we selected the modified Granger and Gray (GG) method^36^, which considered the original Granger and Gray method CR approach to modification. The modified GG model is a universal method that is calibration-free, simple, robust, and uses minimum data, and it is obtained by a comprehensive intercomparison study, and it is not required to pre-estimate PET. The calculation process is as follows^37^:

1 $$ET=2ETw-\mathrm{PET}$$

2 $$ETw=\alpha \frac{\Delta }{\gamma +\Delta }\left({R}_{n}-{G}_{soil}\right)$$

where $\alpha$=1.28, $\Delta$ is the slope of the temperature-saturation vapor pressure curve (kPa℃^−1^), $\gamma$ is the psychrometric constant (kPa℃^−1^), ${R}_{n}$ is the net radiation (mm month^−1^), ${G}_{soil}$ is the soil heat flux (mm month^−1^). where ${R}_{n}$ and ${G}_{soil}$ are estimated using the maximum and minimum temperatures. Granger and Gray (1989) proposed an empirical relationship between PET and ET by defining the relative drying power (D) and the relative ET (G) as:

3 $$D=\frac{{E}_{a}}{{E}_{a}+\left({R}_{n}-{G}_{soil}\right)}$$

4 $$G=\frac{ET}{PET}=\frac{1}{1+{0.028\mathrm{e}}^{8.045D}}$$

5 $$Ea=10.6\times (1+0.5{u}_{2})({e}_{s}-{e}_{a})$$

where ${E}_{a}$ is the drying power of air (mm month^−1^), ${u}_{2}$ is the wind speed of 2 m at ground level, ${e}_{s}$ and ${e}_{a}$ are are the saturation and actual vapor pressures, respectively (mm Hg). From Eqs. (1) and (4), ET can be derived as:

6 $$E{T}_{a}=\frac{2G}{G+1}E{T}_{w}$$

**Figure 10:**
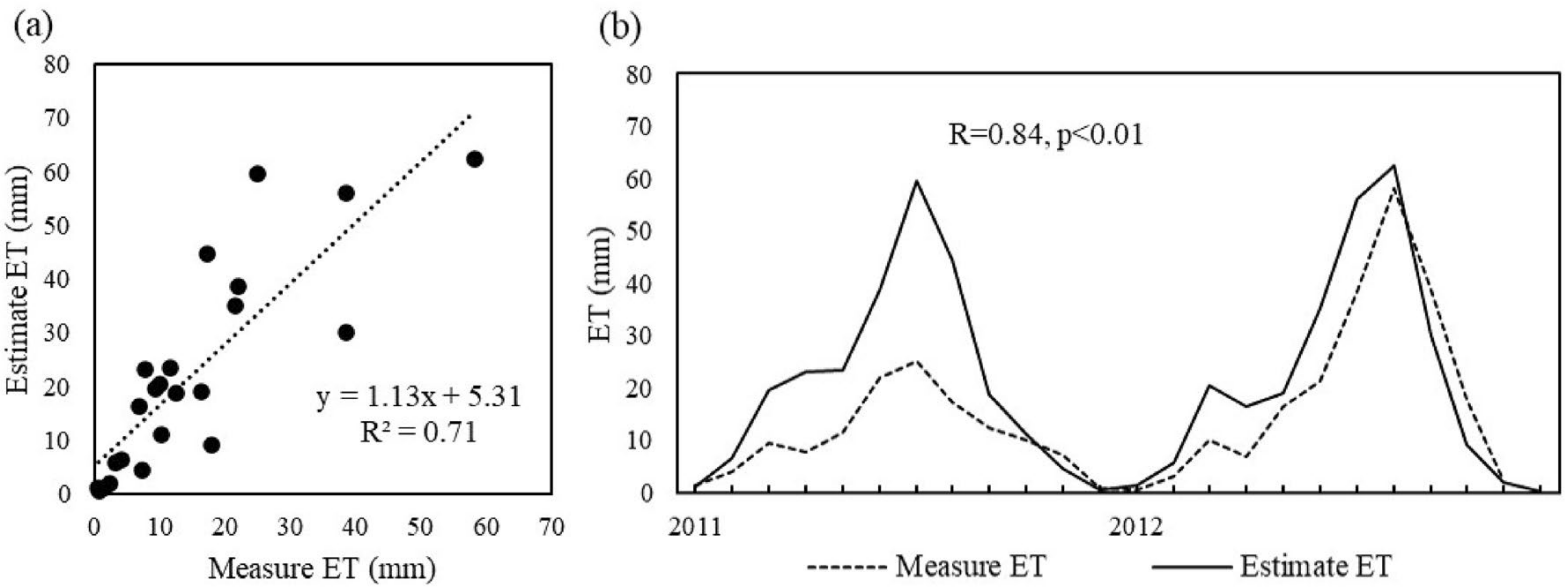
Scatter and trend plots of the measured ET at the Xilinhot station and the estimated ET by the modified GG method.

The ET estimated using meteorological data from the Xilinhot station is compared with the measured ET (Fig. 10). It is show that the estimated ET fitted well with the measured ET (Fig. 10a, R^2^ = 0.71). The two data were significantly correlated with similar trends (Fig. 10b, R = 0.84, *p* < 0.01), with only errors in the high and low values, indicating some reliability of the estimated ET.

### Drought indices selection and calculation

In this study, we used the standardized precipitation evapotranspiration index (SPEI, P-PET), the standardized evapotranspiration drought index (SEDI, ET/PET), and the standardized soil moisture index (SSI, 0-10 cm SM) to represent meteorological drought, ecohydrological drought, and soil drought, respectively. SPEI reflects changes in surface water balance and is considered most suitable for monitoring meteorological drought in the context of global warming^38^. SSI assesses soil moisture deviations at different times and can reflect differences in soil moisture stability from normal levels^39^. ET is an important component of the hydrological cycle, a key process of material and energy exchange in ecosystems, and it is commonly used to estimate ecohydrological drought indices. The evaporative stress index (ESI), for example, is defined as the ratio of ET to PET. A relatively low ESI indicates water limitation to plants, and the actual rate is way below the PET. In contrast, a relatively high ESI indicates freely available water with the AET rate approaching or close to the PET^40^.The ESI is used to assess water stress using hydrological and ecological properties^41^. To compare different types of droughts, we used the standardized procedure for calculating SPEI to calculate the standardized drought index of ESI, i.e., the standardized evapotranspiration drought index (SEDI). The calculation of the standardized drought index requires fitting an appropriate parametric probability distribution, and choosing incorrect probability distributions may lead to inaccurate drought index values and bias the identification of drought events^42^. We used the Fitter library for python 3.9 to evaluate the most suitable distribution types for monthly-scale P-PET, ET/PET, and SM based on Root Mean Square Error (RMSE) and Akaike information criterion (AIC), respectively. We selected gamma distribution, norm distribution, logistic distribution, generalized extreme value (GEV) distribution, Pearson type III (PT-III) distribution, and pareto distribution to fit P-PET, ET/PET, and SM, respectively. The results showed that the PT-III distribution was the most suitable distribution for further calculation of SPEI, which was similar to the study of Wang et al.^43^, who indicated that the PT-III distribution was a reliable distribution for calculating SPEI in the Chinese region:

7 $$F\left(x\right)=\frac{{\beta }^{\alpha }}{\Gamma \left(\alpha \right)}{\int }_{x}^{\infty }{\left(x-\omega \right)}^{\alpha -1}\cdot {\mathrm{e}}^{-\beta \left(x-\omega \right)}dx$$

where $F\left(x\right)$ means the cumulative distribution function of the Person-III distribution, and $\alpha$, $\beta$ and $\omega$ represent the 3 parameters of the distribution. We used energy-balance-based FAO-56 Penman–Monteith equation^44^ to estimate PET (mm d^−1^):

8 $$PET=\frac{0.408\Delta \left({R}_{n}-{G}_{soil}\right)+\gamma \frac{900}{T+273}{u}_{2}({e}_{s}-{e}_{a})}{\Delta +\gamma (1+0.34{u}_{2})}$$

where ${R}_{n}$ and ${G}_{soil}$ are net radiation and soil flux on the ground (MJ m^−2^ d^−1^), $T$ is average daily air temperature (℃), ${e}_{s}$ and ${e}_{a}$ are the saturation and actual vapor pressures (kPa), ${u}_{2}$ is wind speed at 2 m above the ground (m s^−1^), $\Delta$ is the slope of the temperature-saturation vapor pressure curve (kPa℃^−1^), $\gamma$ is the psychrometric constant (kPa℃^−1^).

The Gamma distribution was used as the most suitable distribution for calculating SEDI and SSI:

9 $$G\left(x\right)=\frac{1}{{\beta }^{\alpha }\Gamma \left(\alpha \right)}{x}^{\alpha -1}{\mathrm{e}}^{-\frac{x}{\beta }}, x>0$$

where $G\left(x\right)$ means the cumulative distribution function of the gamma distribution, and $\alpha$, $\beta$ represent the parameters of the distribution. Lastly, the standardization process is used to calculate the SPEI (SEDI and SSI) value by converting the fitted distribution function $F\left(x\right)$($G\left(x\right)$) to the standard normal distribution a mean of 0 and a standard deviation of 1. The SPEI (SEDI and SSI) value is derived as the normalized value of $F\left(x\right)$($G\left(x\right)$).

SPEI is a drought index that contains multiple time scales (denoted as SPEI-n, n is the time scale), with 3–6 months indicating seasonal drought and above 12-month scale indicating interannual drought^6,45^. The region frequently experiences seasonal drought, and the growing season is in 6 months (April-September). Therefore, considering the frequent seasonal droughts in the study area, with a 6-month growing season (April-September), we selected a 12-month scale to analyze the interannual variation of different types of droughts, as well as the cascading effects among different types of droughts using a 6-month scale. We classified the drought severity levels of the three drought indices into consistent (Table 2).

**Table 2:** Drought severity classification of three drought indices (DI represents SPEI, SEDI, or SSI).

Drought grade	Drought indices (DI) Value
No drought	DI > −0.5
Mild drought	−1.0 < DI ≤ −0.5
Moderate drought	−1.5 < DI ≤ −1.0
Severe drought	−2.0 < DI ≤ −1.5
Extreme drought	DI ≤ -2.0

### The Mann–Kendall trend and mutation test method

The Mann–Kendall trend test is a nonparametric method which used to determine the significance of trends in a time series. The formula is as follows:

10 $$S=\sum_{k=1}^{n-1}\sum_{j=k+1}^{n}sgn\left({X}_{j}-{X}_{k}\right)$$

The range of values of $sgn(x)$:

11 $$\mathrm{sgn}\left({x}_{j}-{x}_{k}\right)=\left\{\begin{array}{c}1 \left({x}_{j}-{x}_{k}\,>\,0\right)\\ 0 \left({x}_{j}-{x}_{k}\,=\,0\right)\\ -1 \left({x}_{j}-{x}_{k}\,<\,0\right)\end{array}\right.$$

where ${x}_{j}$ and ${x}_{k}$ are time series data, S is normally distributed with mean 0. The variance $Var\left(S\right)=n\left(n-1\right)\left(2n+5\right)/18$. When n > 10, the standard normal statistic variable Z is:

12 $$Z=\left\{\begin{array}{c}\frac{s-1}{\sqrt{\mathrm{Var}\left(s\right)}} \quad s\,>\,0\\ 0 \quad s\,=\,0\\ \frac{s+1}{\sqrt{\mathrm{Var}\left(S\right)}} \quad s\,<\,0\end{array}\right.$$

Therefore, at a given confidence level α, if $\left|Z\right|\ge {Z}_{1-\alpha /2}$, the original hypothesis is not acceptable, and there is a significant increasing or decreasing trend of the time series data at the α confidence level. When the statistic $Z>0$, the series has an increasing trend; when $Z<0$, the series has a decreasing trend. In this study, when $\left|Z\right|$ is greater than 1.96 and 2.58, it indicates that the trend has passed the significance test with a reliability of 95% and 99% respectively.

### Pearson correlation coefficient (PCC)

We analyzed the seasonal propagation relationship between different kinds of drought using the maximum Pearson correlation coefficient (MPCC). This method has been applied to several similar studies and can effectively represent the propagation time between different drought types^15,46^. The PPC formula is as follows:

13 $$PPC=\frac{{\Sigma }_{\mathrm{i}=1}^{n}\left({x}_{i}-\overline{x }\right)\left({y}_{i}-\overline{y }\right)}{\sqrt{{\Sigma }_{\mathrm{i}=1}^{n}{\left({x}_{i}-\overline{x }\right)}^{2}}\sqrt{{\Sigma }_{\mathrm{i}=1}^{n}{\left({y}_{i}-\overline{y }\right)}^{2}}}$$

where ${x}_{i}$ and ${y}_{i}$ represent two time series. The value of PPC is between -1 and 1, where $PPC>0$ indicates positive correlation, $PPC<0$ indicates negative correlation, and $PPC=0$ indicates no correlation.

### Partial least squares structural equations modeling (PLS-SEM)

Structural Equation Model (SEM) is a multivariate statistical model, which is widely used in social science investigations to test and evaluate multivariate causal relationships. Partial least squares structural equations modeling (PLS-SEM) is a nonparametric multivariate method based on iterative ordinary least squares (OLS) regression, which operates more similarly to multiple regression analysis^47^. Compared with traditional SEM, PLS-SEM can be applied to study data with non-normal distribution and small sample size, and it can also deal with problems that often occur in the modeling process, such as anomalous data characteristics and highly complex models. PLS-SEM mainly defines a principal component structure by fitting a linear relationship of variables and then uses the principle of regression analysis to test the explanatory relationship between principal components^48^. It can predict how much variance change is explained by exogenous variables to endogenous variables and detect whether the causal relationship is significant. PLS-SEM is suitable in highly complex exploratory models, which can also be used to test the explored causal relationships. Therefore, in this study, we selected PLS-SEM to explore the cascading effects between different types of droughts. The specific modeling process is described in Supplementary.

## Supplementary Information

Supplementary Information.

## Data Availability

The meteorological observation data are available online (http://data.cma.cn/en). The Global Land Data Assimilation System (GLDAS) data sets (version 2.0 and 2.1) are free available online (https://disc.gsfc.nasa.gov/datasets?keywords=GLDAS). The GLOBMAPLAI V3 are free available online (https://www.resdc.cn/data.aspx?DATAID=336).
