# Supplementary material for: Cascading effects of drought in Xilin Gol temperate grassland, China
Source: Sci Rep. 2023 Jul 5;13:10926. doi: 10.1038/s41598-023-38002-2 (PMC10322895; doi:10.1038/s41598-023-38002-2)
Supplement: Supplementary file 1 — Supplementary Information. [file 41598_2023_38002_MOESM1_ESM.docx]

**Cascading effects of Drought in** **Xilin Gol Temperate Grasslands, China**

Jingzhao Ma^1,2^, Jingmin Gao^1,3*^

^1^Collaborative Innovation Center on Forecast and Evaluation of Meteorological Disasters (CIC-FEMD) / Jiangsu Key Laboratory of Agricultural Meteorology, Nanjing University of Information Science and Technology, Nanjing, P. R. China.

^2^Longchuan Meteorological Bureau, Longchuan 517300, Guangdong, China.

^3^Emergency Management College, Nanjing University of Information Science and Technology, Nanjing, P. R. China.


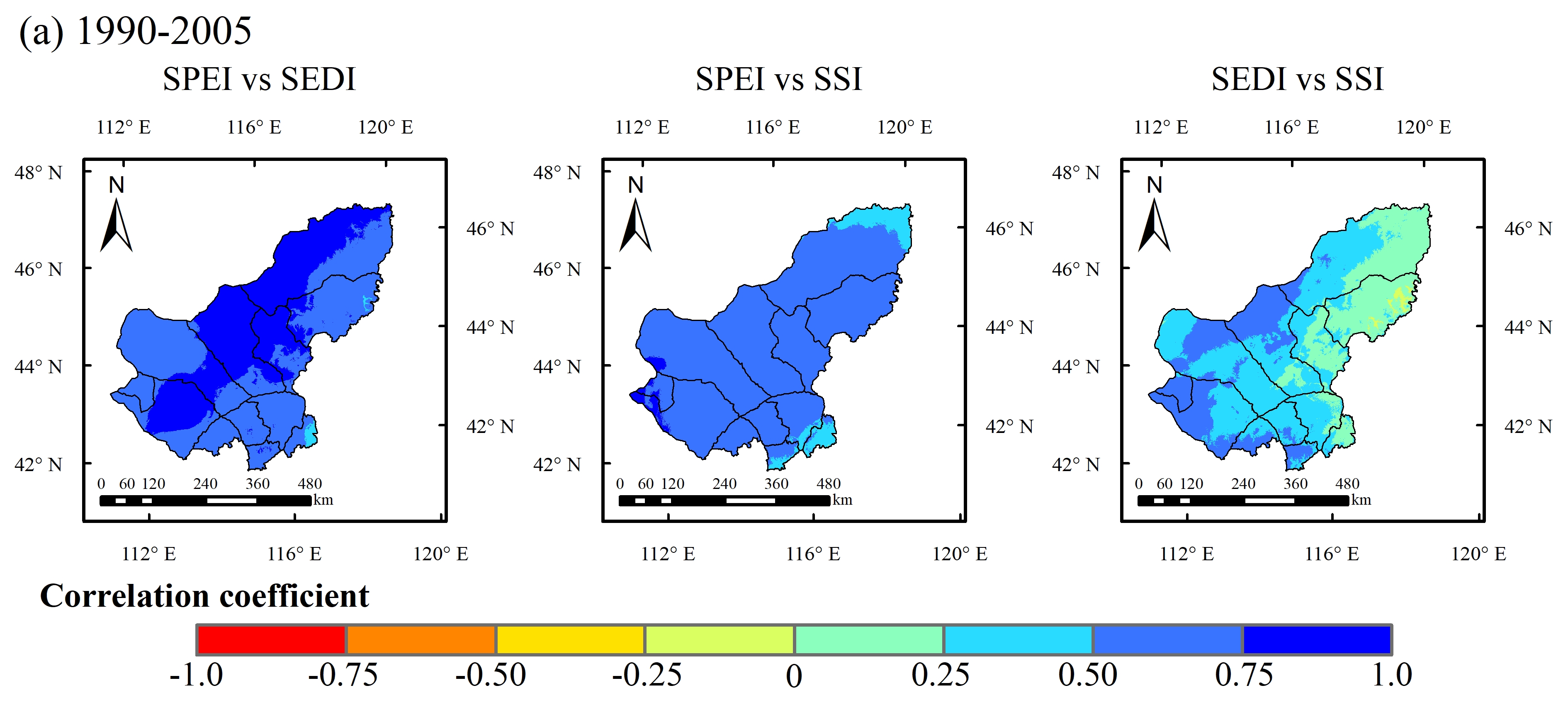


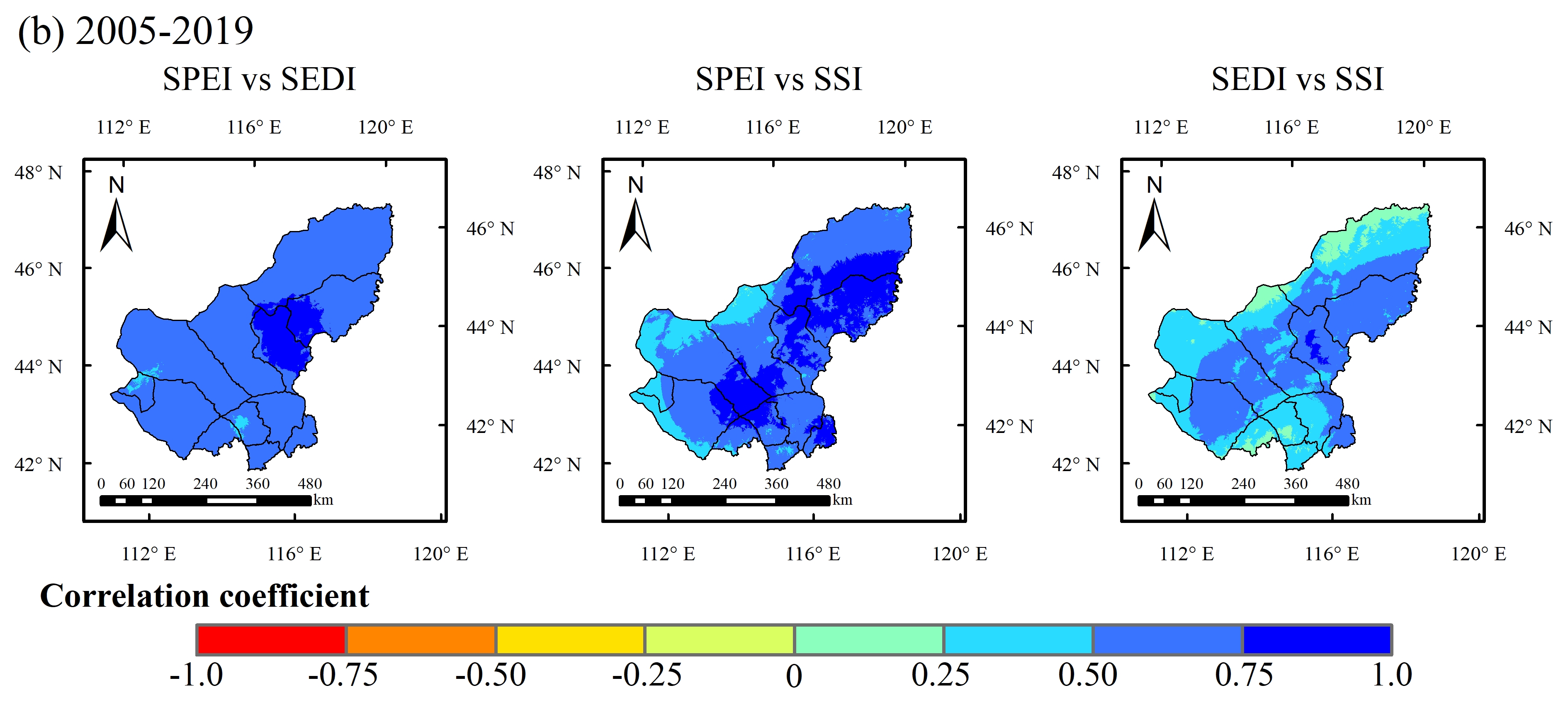


Figure S1 Correlation of Annual meteorological drought, ecohydrological drought and soil drought (a. 1990-2005 and b. 2005-2019). (The figure was generated by ArcGIS 10.6 software, https://desktop.arcgis.com/en/)


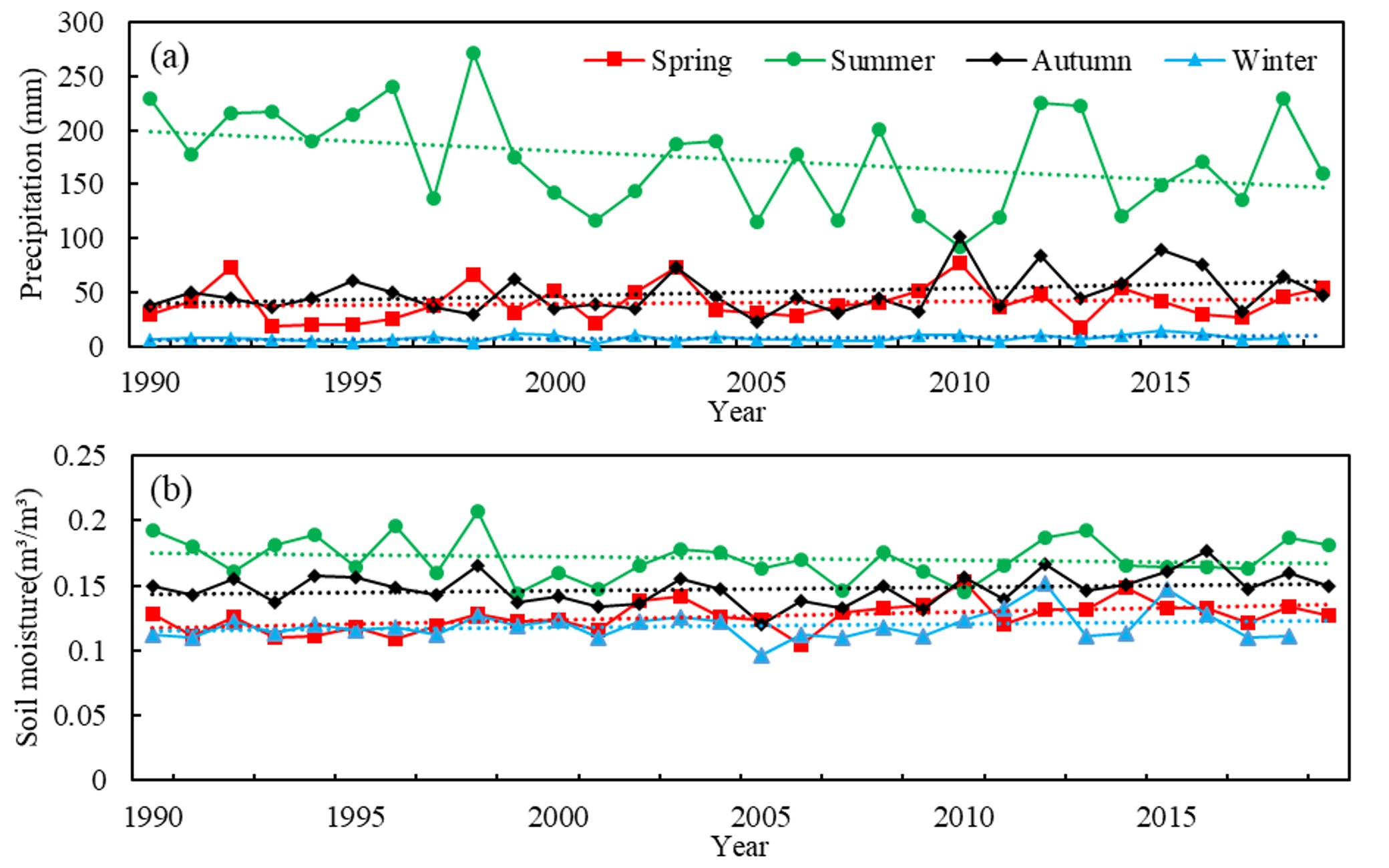


Figure S2 Seasonal trends in (a) precipitation and (b) soil moisture.


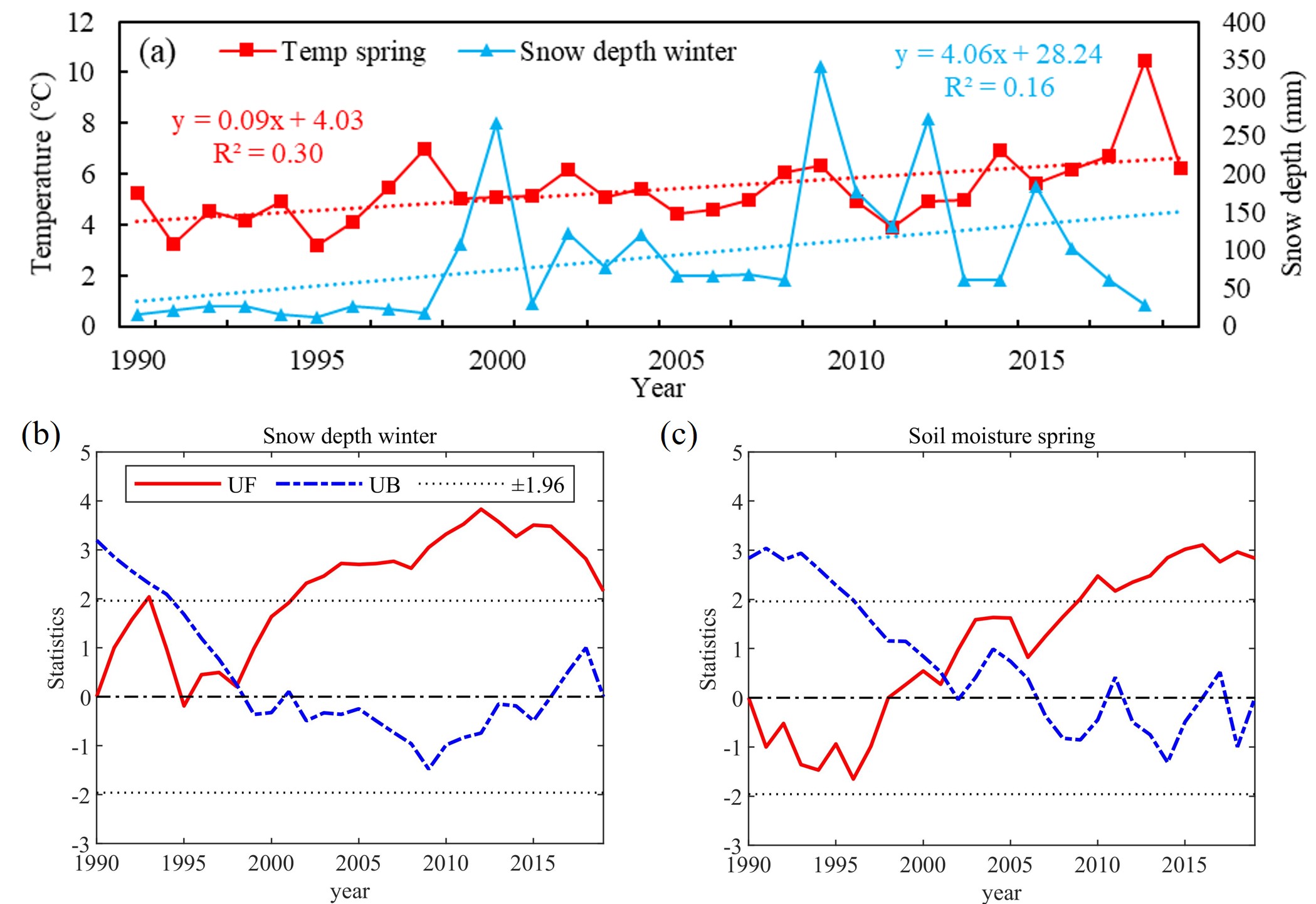


Figure S3 Temporal trends of spring temperature and winter snow depth(a), and winter snow depth(b) and spring soil moisture(c) Mann-Kendall (MK) mutation test.


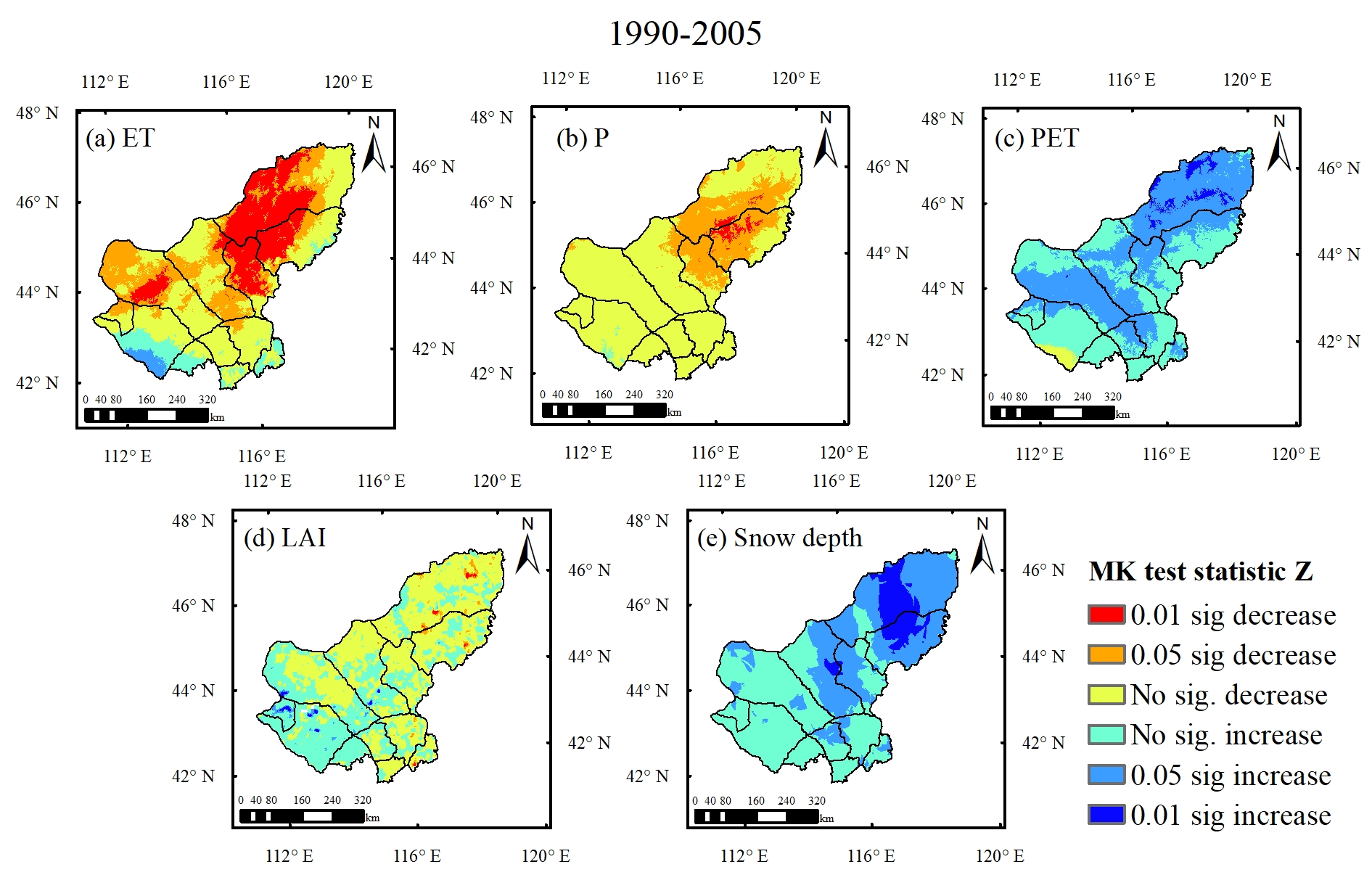


Figure S4 Spatial trend distribution of biophysical drivers (a)-(e). (1990-2005). (The figure was generated by ArcGIS 10.6 software, https://desktop.arcgis.com/en/)


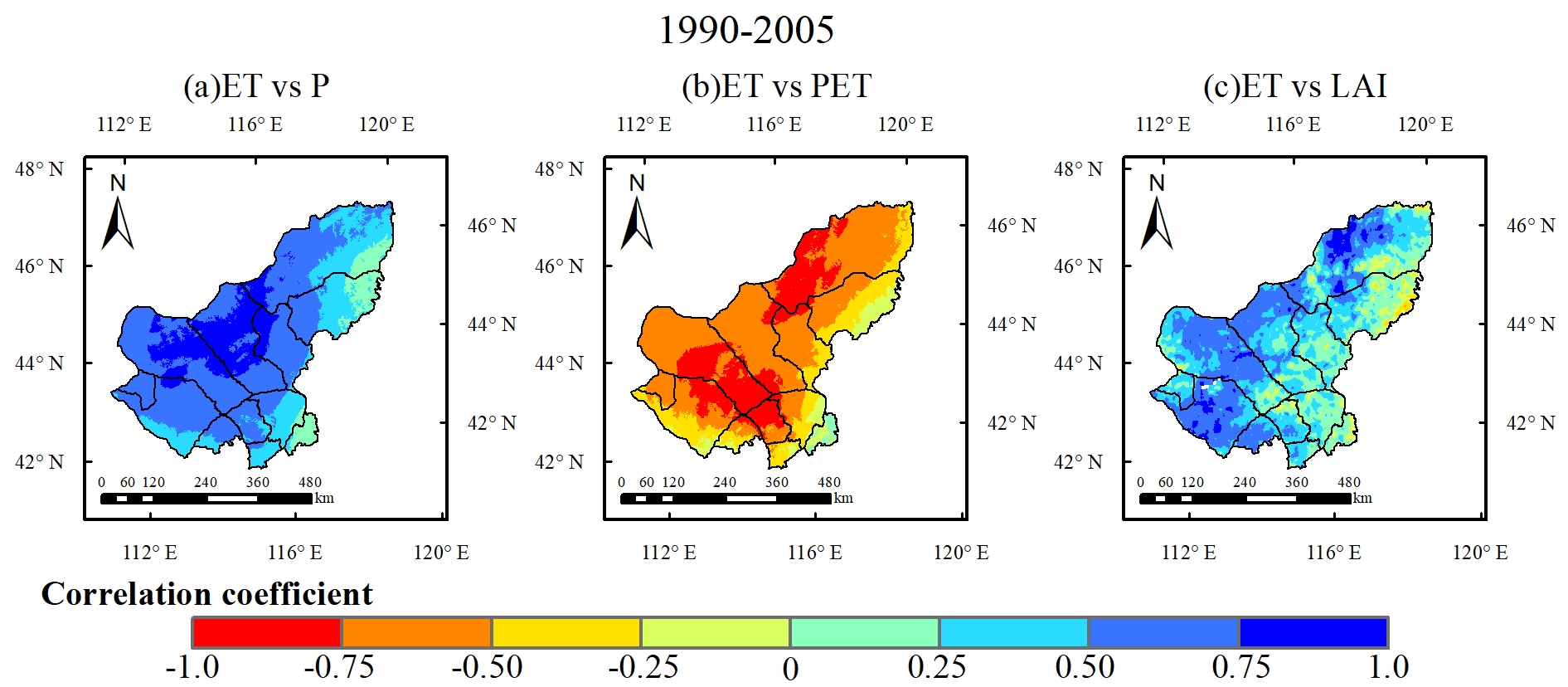


Figure S5 Correlation of Annual biophysical drivers (1990-2005). (The figure was generated by ArcGIS 10.6 software, https://desktop.arcgis.com/en/)


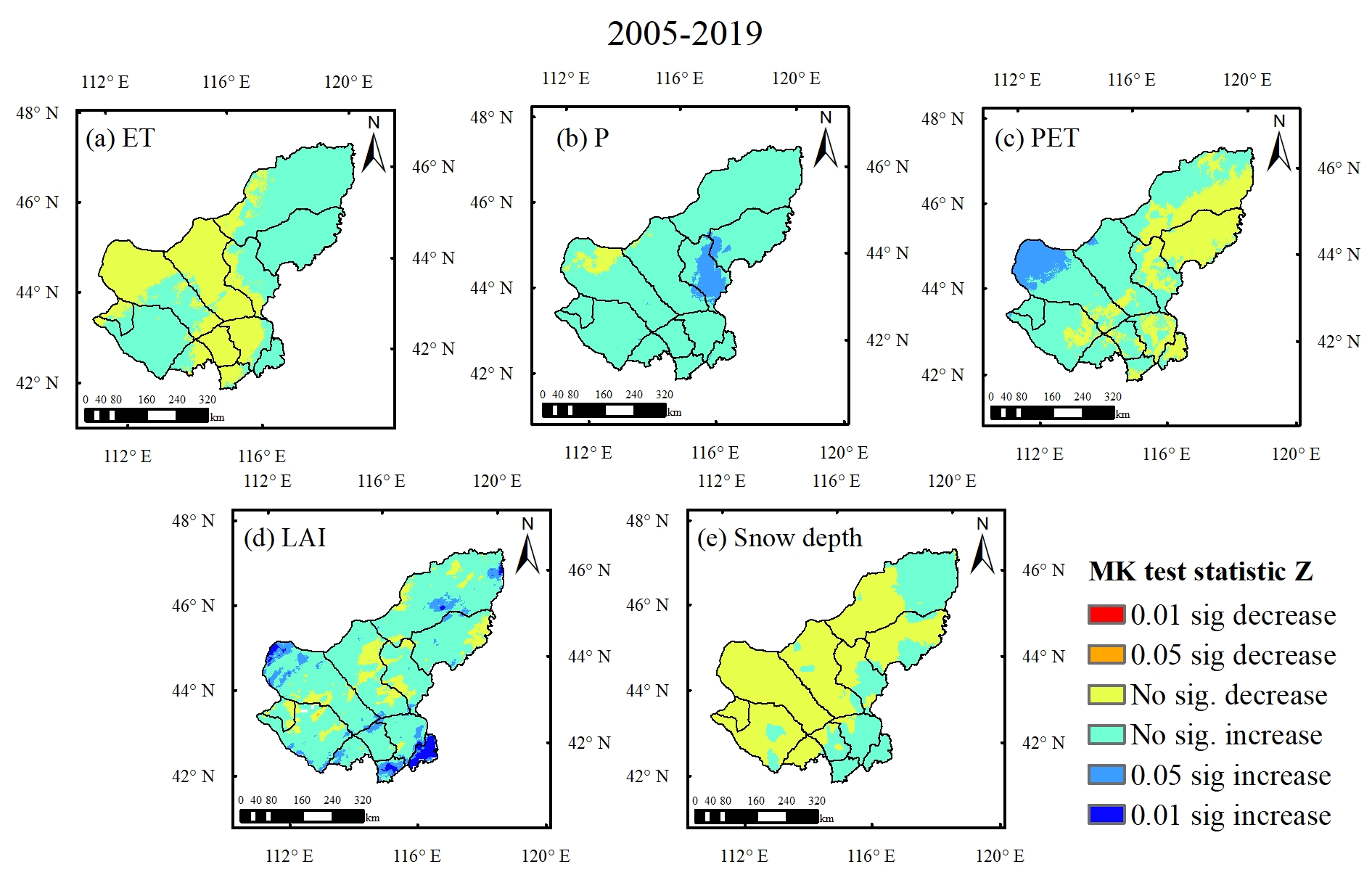


Figure S6 Spatial trend distribution of biophysical drivers (a)-(e). (2005-2019). (The figure was generated by ArcGIS 10.6 software, https://desktop.arcgis.com/en/)


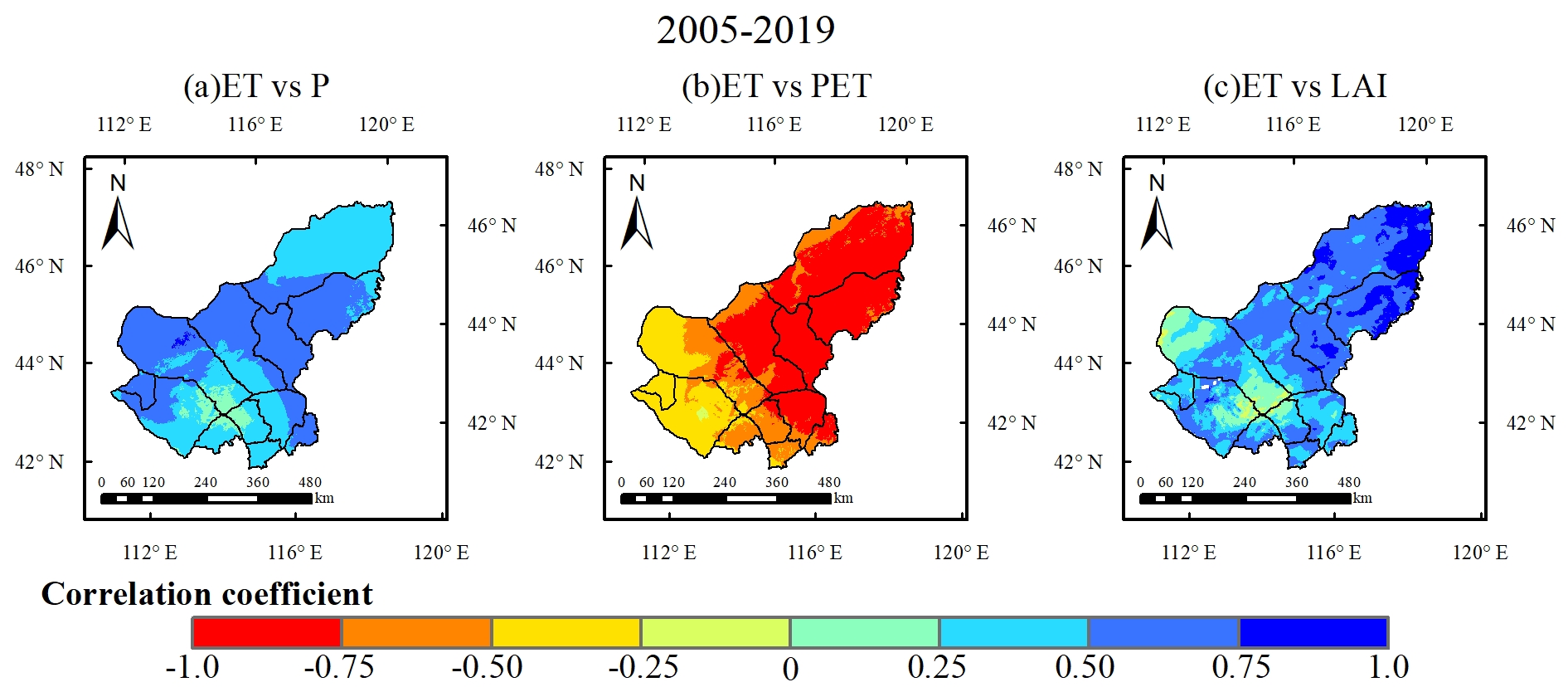


Figure S7 Correlation of Annual biophysical drivers (2005-2019). (The figure was generated by ArcGIS 10.6 software, https://desktop.arcgis.com/en/)

#### PLS-SEM

We used the PLS-SEM method to construct a causal model between drought types to further detect the causal relationships (cascade effects) among different drought types under the influence of snow and vegetation. We divided the regions for modeling based on the climatic characteristics of the study area, i.e., eastern, southern, central, and northwestern (Table S1). In applying PLS-SEM, we referred to the important steps recommended by Hair (2014) to construct the model. We performed data collection and processing, specification of the measurement model and structural model, estimation of the model, and evaluation of the results ^1^.

Table S1 Xilin Gol regional divisions and the corresponding Banners and Meteorological stations

| Region | Banners and Meteorological stations |
| --- | --- |
| East | East Ujimqin, West Ujimqin |
| South | Zhenglan, Zhengxiangbai, Duolun, Taibus |
| Centre | Naranbulag, Abag, XilinHot |
| Northwest | Sonidzuo, Sonidyou, Zhurihe, Erenhot |

#### Model specification

First, the path model was created by connecting variables and structures according to theory. The process focuses on building measurement models and structural models. In general, meteorological droughts due to precipitation shortages are the initiators that cause other drought types. Therefore, the relationship between meteorological drought, soil drought, and ecohydrological drought was determined. The meteorological drought hazard (MDH) was considered as an exogenous latent variable, and the soil drought hazard (SDH) and ecohydrological drought hazard (EDH) were considered as endogenous latent variables, pointing snow and vegetation to soil drought and ecohydrological drought. Hypothetical relationships between exogenous latent variables and endogenous latent variables were established to determine the structural model. Suitable observed variables, representing the respective latent variables, were selected to determine the measurement model. In this study, the drought indicators on a 6-month time scale were selected to represent the corresponding drought types, and the model for the eastern region was selected to demonstrate the testing process. Where M1, M2 represent meteorological drought indicators SPEI-6 for different stations, E1, E2 represent ecohydrological drought indicators SEDI-6, and S1, S2 represent soil drought indicators SSI-6 (Fig. S8). The main research questions are: (1) the causal characteristics of seasonal drought; (2) how do snow and vegetation affect (directly OR indirectly) seasonal drought? Each latent variable with its corresponding observed variable is shown in Table S2.

Table S2 Latent variables and their corresponding measured variables in study model

| **Latent variable** | **observable indicators** |
| --- | --- |
| Meteorological drought Hazard (MDH) | Meteorological station SPEI-6 |
| Ecohydrological drought Hazard (EDH) | Meteorological station SEDI-6 |
| Soil drought Hazard (SDH) | Meteorological station SSI-6 |
| Vegetation | LAI |
| Snow | Snow depth |

(Note: In order to comply with the definition of latent variables, all drought indicators were multiplied by -1, i.e., the larger the value of the indicator, the more severe the drought condition. To unify the sample size, LAI and Snow depth were treated as 6 months cumulative. Sample size = 355.)


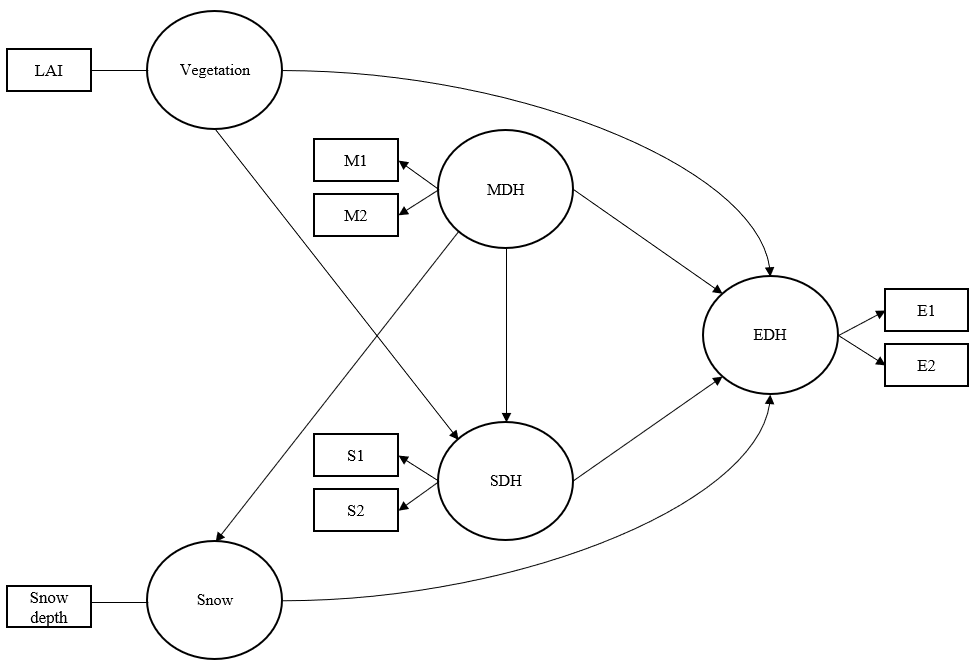


Figure S8 A causal path model between different drought types (east).

#### The measurement model evaluation

After the path model was determined, the latent variables in the measurement model (outer models) were tested for reliability and validity. There are two different types of indicators in PLS-SEM, i.e., reflective indicators and formative indicators. These two types of indicators have different assessment methods, so it is important to distinguish between them when evaluating measurement models. Only reflective indicators exist for the measurement model in this study. Therefore, we need to validate it examine reliability, convergent validity and discriminant validity. We selected the eastern model for demonstration. Table S3 shows the results of the test of the measurement model, where the factor loadings, Cronbach's Alpha, Composite Reliability (CR), and Average Variance Extracted (AVE) of the assessed indicator observation variables reached the thresholds of 0.7, 0.7, 0.7, and 0.5 above the threshold, indicating that the latent variables of the study's measurement model have intrinsically consistent reliability and convergent validity. This provides assistance in determining the assumed relationships for the structural model.

Table S3 Assessment of Measurement model reliability and validity

| **Latent variable** | **observable indicators** | Loadings | CR (>0.7) | Cronbach’s Alpha (>0.7) | AVE  (>0.5) |
| --- | --- | --- | --- | --- | --- |
| MDH | M1  M2 | 0.931  0.937 | 0.932 | 0.853 | 0.872 |
|  |  |  |  |  |  |
| EDH | E1  E2 | 0.951  0.934 | 0.941 | 0.876 | 0.889 |
| SDH | S1  S2 | 0.972  0.971 | 0.971 | 0.940 | 0.944 |
| Vegetation | LAI | 1.0 | 1.0 | 1.0 | 1.0 |
| Snow | Snow depth | 1.0 | 1.0 | 1.0 | 1.0 |

Discriminant validity means the difference between the constructs and other constructs. Two assessment methods were used to examine the discriminant validity of the constructs. The first one is cross loadings, which requires that the cross-loadings of the indicators of the constructs must be higher than all other constructs. The second is the Fornell and Larcker (1981) criterion, which requires that the root value of the AVE for each construct should be higher than the correlation with any other construct. Table S4 shows the cross-loadings of the indicator variables in the reflective measurement model for the eastern model, and it can be noted that the highest loadings for each indicator are at specific constructs.

Table S4 Cross loading of indicator variables in the measurement model

|  | M | E | S | Snow | Vegetation |
| --- | --- | --- | --- | --- | --- |
| E1 | 0.765 | **0.951** | 0.361 | -0.072 | -0.123 |
| E2 | 0.663 | **0.934** | 0.325 | -0.06 | -0.096 |
| M1 | **0.931** | 0.669 | 0.6 | -0.18 | -0.137 |
| M2 | **0.937** | 0.75 | 0.576 | -0.159 | -0.148 |
| S1 | 0.618 | 0.361 | **0.972** | -0.227 | -0.165 |
| S2 | 0.605 | 0.348 | **0.971** | -0.259 | -0.17 |
| LAI | -0.153 | -0.117 | -0.172 | -0.463 | **1** |
| Snow depth | -0.181 | -0.07 | -0.25 | **1** | -0.463 |

Table S5 shows the Fornell-Larcker criterion for the indicator variables of the reflectivity measurement model. The results show that the AVE root values of each construct are the maximum values in the ranks of the table, i.e., the AVE root values are greater than the correlation coefficients of the latent variables, thus, it can be shown that each construct has discriminant validity.

Table S5 The Fornell-Larcker criterion of indicator variables in the measurement model

|  | E | M | Snow | S | | Vegetation |
| --- | --- | --- | --- | --- | --- | --- |
| E | **0.94** |  |  |  |  | |
| M | 0.76 | **0.93** |  |  |  | |
| Snow | -0.07 | -0.18 | **1** |  |  | |
| S | 0.37 | 0.63 | -0.25 | **0.97** |  | |
| Vegetation | -0.12 | -0.15 | -0.46 | -0.17 | **1** | |

#### The structural model evaluation

The validation in the previous section shows that the measurement model in this study has reliability and validity. The next step is to assess the hypothesized relationships of the structural model (inner model). First, collinearity needs to be assessed both internally and externally for each latent variable. If there is collinearity between the constructs, it will lead to bias in the path coefficients. The variance inflation factor (VIF) is an indicator for the assessment of collinearity. Hair et al. (2014) suggests that the absence of multicollinearity between variables can only be indicated when VIF < 5. The VIF values of all latent variables inside and outside the model constructed in this study are less than 5, so it can be considered that there is no significant multicollinearity in the variables of the model (Table S6, S7).

Table S6 Observed variable VIF value

|  | VIF |
| --- | --- |
| E1 | 2.545 |
| E2 | 2.545 |
| M1 | 2.239 |
| M2 | 2.239 |
| S1 | 4.698 |
| S2 | 4.698 |
| LAI | 1 |
| Snow depth | 1 |

Table S7 Latent variable VIF value

|  | E | M | Snow | S | Vegetation |
| --- | --- | --- | --- | --- | --- |
| E |  |  |  |  |  |
| M | 1.67 |  | 1 | 1.116 | 1 |
| Snow | 1.492 |  |  | 1.387 |  |
| S | 1.799 |  |  |  |  |
| Vegetation | 1.445 |  |  | 1.373 |  |

The assessment of model quality is based on its ability to predict endogenous latent variables. The following indicators contribute to the assessment: coefficient of determination (R^2^), cross-validated redundancy (Q^2^), path coefficients (PC), and the effect size (f^2^), as well as for detecting model fitness: Standardized Root Mean Square Residual (SRMR).

The R² is the accuracy measure of the model prediction. In other words, it represents the combined effect of exogenous latent variables on endogenous latent variables. The R² ranges from 0 to 1, and higher values indicate that the model has higher prediction accuracy. Generally, R² is 0.75, 0.50, and 0.25 to describe substantial, moderate, or weak levels of predictive accuracy ^2^. The Q^2^ is a way to assess the predictive relevance of the structural model, indicating whether endogenous latent variables can be predicted. A Q² larger than 0 means that there is a prediction correlation, and the larger the Q^2^, the higher the prediction accuracy of the model. The t-value is used to detect the significance of the path coefficients. When the t-value is larger than 1.96, it indicates 0.05 level of significance, and when the t-value is larger than 2.58, it indicates 0.01 level of significance. The t-value can be used to determine whether the causal relationship between variables is significant or not. The effect size (f^2^) assesses the effect of exogenous latent variables on endogenous latent variables (contribution), and f² values of 0.35, 0.15, and 0.02 are generally considered to indicate large, medium, and small effects, respectively. SRMR is used to test the fitness of the model, and values less than 0.1 are acceptable, while a more stringent criterion is an SRMR of less than 0.8.

Table S8 shows the test results of the structural model cross-validated redundancy, which shows that the Q² of each endogenous latent variable of the model is larger than 0, indicating the existence of predictive correlation of exogenous latent variables to endogenous latent variables. Table S9 shows the R^2^ for the eastern region, it can be concluded that the endogenous latent variable (ecohydrological drought hazard) has a moderate degree of explanation. Table S10 demonstrates the size of the effects between the various constructs, and it can be concluded that there is a large effect of meteorological drought on both ecohydrological drought (f²=1.17) and soil drought (f²=0.497). Table S11 demonstrates the results of the significance tests of the path coefficients of the eastern regional structural model. The results show that meteorological drought has significant effects on ecohydrological drought, soil drought, vegetation and snow.

**Table S8** Structural model cross-validated redundancy test results

|  | SSO | SSE | Q² (=1-SSE/SSO) |
| --- | --- | --- | --- |
| E | 710 | 335.692 | 0.527 |
| M | 710 | 710 |  |
| Snow | 355 | 344.832 | 0.029 |
| S | 710 | 415.501 | 0.415 |
| Vegetation | 355 | 348.046 | 0.02 |

**Table S9** Determinable coefficients (R^2^) of endogenous latent variables (East)

|  | R^2^ |
| --- | --- |
| E | 0.602 |
| S | 0.033 |
| Snow | 0.444 |
| Vegetation | 0.023 |

**Table S10** Effect size f²values between the latent variables in the structural model (East)

|  | E | M | Snow | S | Vegetation |
| --- | --- | --- | --- | --- | --- |
| E |  |  |  |  |  |
| M | **1.17** |  | 0.034 | **0.497** | 0.024 |
| Snow | 0.004 |  |  | 0.076 |  |
| S | 0.043 |  |  |  |  |
| Vegetation | 0 |  |  | 0.052 |  |

**Table S11** Structural model path coefficient significance test results (East)

|  | Original Sample (O) | Sample Mean (M) | Standard Deviation (STDEV) | T Statistics (\|O/STDEV\|) | Sig. | P Values |
| --- | --- | --- | --- | --- | --- | --- |
| M-> E | **0.882** | 0.884 | 0.037 | 23.908 | ** | 0.000 |
| M -> Snow | **-0.181** | -0.18 | 0.059 | 3.089 | ** | 0.003 |
| M -> S | **0.555** | 0.555 | 0.042 | 13.137 | ** | 0.000 |
| M -> Vegetation | **-0.153** | -0.151 | 0.05 | 3.025 | ** | 0.003 |
| Snow -> E | **0.051** | 0.05 | 0.047 | 1.075 | NS | 0.287 |
| Snow -> S | **-0.242** | -0.242 | 0.056 | 4.313 | ** | 0.000 |
| S -> E | **-0.176** | -0.178 | 0.048 | 3.636 | ** | 0.000 |
| Vegetation -> E | **0.011** | 0.011 | 0.039 | 0.28 | NS | 0.783 |
| Vegetation -> Soil | **-0.199** | -0.198 | 0.049 | 4.037 | ** | 0.000 |

(note：*p＜0.05，**p＜0.01，NS= not significant. )

Mediating effects refer to the fact that the relationship between the effects of variables is not a direct causal chain, but arises indirectly through one or more of the variables. Using the Eastern model as an example, the indirect effect of Vegetation on EDH through SDH is called the mediating effect (Fig. S9). Fig. S9 shows the specific test steps. This study uses the variance accounted for (VAF) test, which refers to the percentage of indirect effects and total effects. Generally, VAF < 20% indicates no mediation; 20% ≤ VAF ≤ 80% indicates the presence of partial mediation, and VAF > 80% indicates full mediation. The formula is:

$VAF=\frac{P2\times P3}{P2\times P3+P1}\times100\%$ (B.1)


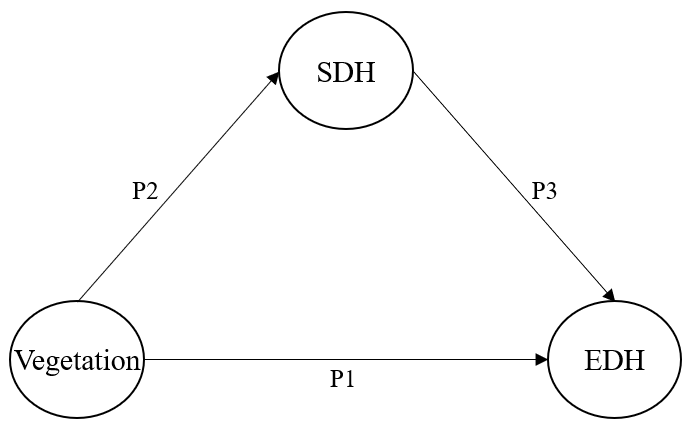

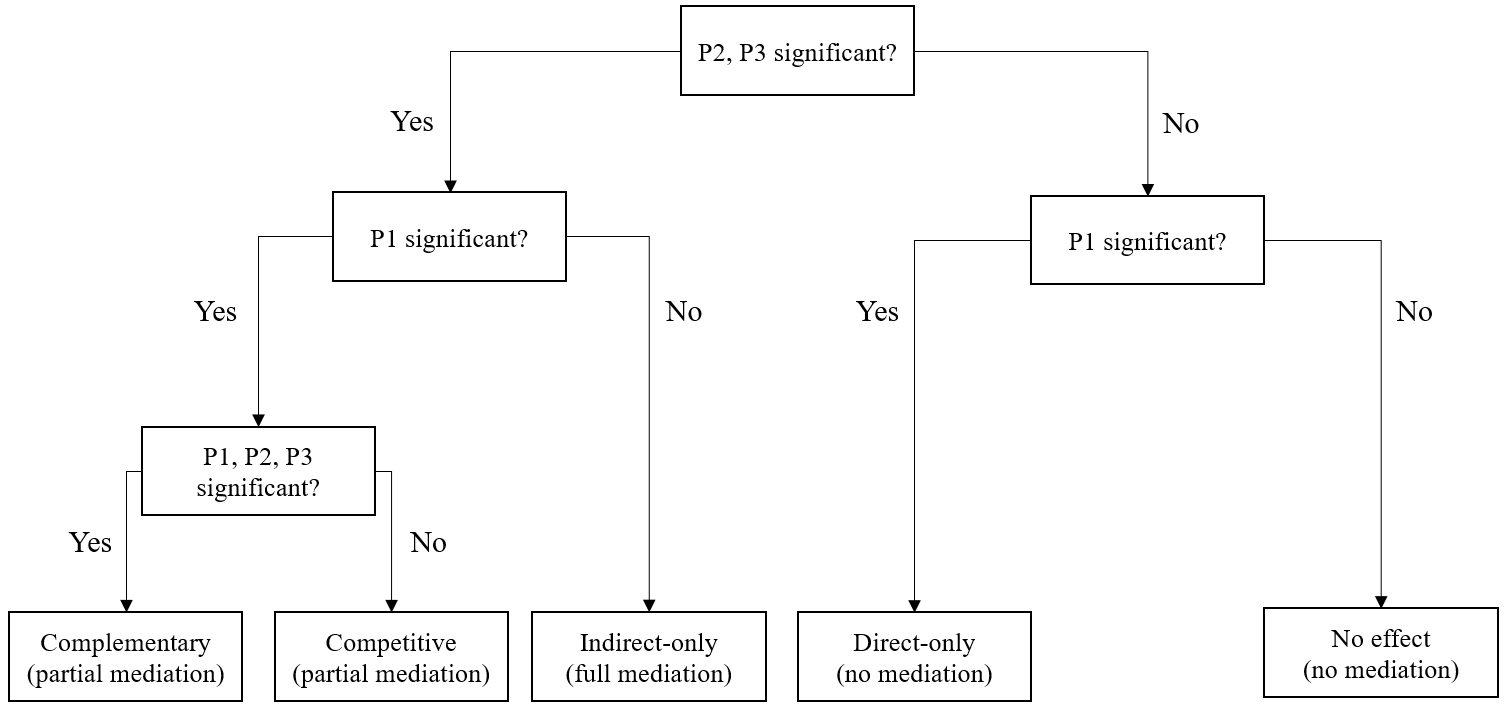


Figure S9 Mediation analysis procedure and test of indirect effects

According to Fig. A.9 steps, the relationship of latent variables of the eastern model was tested for mediation effects using VAF values. The VAF value was calculated to be 76.1%, which is between 20% and 80% (Table S11, eq. B.1). It shows that the influence of vegetation on ecohydrological drought in the eastern region is partially mediated, and it is indirectly influencing ecohydrological drought by affecting soil drought.

Table S12 shows the model fitness (SRMR) of the four regions. the SRMR values are all less than 0.08, so the PLS-SEM of this study can be considered to have excellent fitness. It can well explain the causal relationship between different drought types under different vegetation types.

The example of the model testing process above is for the model of the eastern region. Similarly, the same steps are performed to test the models for the other three regions (Center, South, and Northwest). The models for each region have excellent fitness (Table S12, SRMR<0.08).

**Table S12** Model fit SRMR

| Region | SRMR (<0.08) |
| --- | --- |
| East | 0.043 |
| South | 0.072 |
| Center | 0.046 |
| Northwest | 0.076 |

**Table S13** Determinable coefficients (R^2^) of endogenous latent variables (South, northwest and center)

|  | R^2^(South) | R^2^(Northwest) | R^2^(Center) |
| --- | --- | --- | --- |
| E | 0.464 | 0.577 | 0.624 |
| S | 0.041 | 0.055 | 0.028 |
| Snow | 0.336 | 0.387 | 0.413 |
| Vegetation | 0.012 | 0.038 | 0.034 |

**Table S14** Effect size f² values between the latent variables in the structural model (South, northwest and center)

|  | f^2^(South) | f^2^ (Northwest) | f^2^ (Center) |
| --- | --- | --- | --- |
| M-> E | **0.639** | **0.765** | **1.209** |
| M -> Snow | 0.043 | 0.058 | 0.029 |
| M -> S | **0.202** | **0.335** | **0.270** |
| M -> Vegetation | 0.012 | 0.039 | 0.035 |
| Snow -> E | 0.055 | 0.004 | 0.031 |
| Snow -> S | 0.141 | 0.035 | **0.185** |
| S -> E | 0.027 | 0.005 | 0.002 |
| Vegetation -> E | 0.012 | 0.000 | 0.018 |
| Vegetation -> S | 0.072 | 0.077 | 0.115 |

**Table S15** Structural model path coefficient (PC) significance test results (South, northwest and center)

|  | PC(South) | PC(Northwest) | PC(Center) |
| --- | --- | --- | --- |
| M-> E | 0.673** | 0.715** | 0.802** |
| M -> Snow | -0.203** | -0.234** | -0.167** |
| M -> S | 0.385** | 0.492** | 0.421** |
| M -> Vegetation | -0.111* | -0.195** | -0.184** |
| Snow -> E | -0.214** | -0.048 ^NS^ | 0.134** |
| Snow -> S | -0.359** | -0.171** | -0.374** |
| S -> E | -0.147** | 0.057 ^NS^ | 0.038^NS^ |
| Vegetation -> E | -0.096* | 0.016^NS^ | 0.100* |
| Vegetation -> Soil | -0.252** | -0.250** | -0.296** |

(note：*p＜0.05，**p＜0.01，NS= not significant. )

# References

1 F. Hair Jr, J., Sarstedt, M., Hopkins, L. & G. Kuppelwieser, V. Partial least squares structural equation modeling (PLS-SEM). *European Business Review* **26**, 106-121, doi:10.1108/ebr-10-2013-0128 (2014).

2 Hair, J. F., Sarstedt, M., Ringle, C. M. & Mena, J. A. An assessment of the use of partial least squares structural equation modeling in marketing research. *Journal of the Academy of Marketing Science* **40**, 414-433, doi:10.1007/s11747-011-0261-6 (2011).
